# What Caused What? A Quantitative Account of Actual Causation Using Dynamical Causal Networks

**DOI:** 10.3390/e21050459

**Published:** 2019-05-02

**Authors:** Larissa Albantakis, William Marshall, Erik Hoel, Giulio Tononi

**Affiliations:** 1 Department of Psychiatry, Wisconsin Institute for Sleep and Consciousness, University of Wisconsin-Madison, Madison, WI 53719, USA; 2 Department of Mathematics and Statistics, Brock University, St. Catharines, ON L2S 3A1, Canada; 3 Allen Discovery Center, Tufts University, Medford, MA 02155, USA

**Keywords:** graphical models, integrated information, counterfactuals, Markov condition, primary 62-09, secondary 60-J10

## Abstract

Actual causation is concerned with the question: “What caused what?” Consider a transition between two states within a system of interacting elements, such as an artificial neural network, or a biological brain circuit. Which combination of synapses caused the neuron to fire? Which image features caused the classifier to misinterpret the picture? Even detailed knowledge of the system’s causal network, its elements, their states, connectivity, and dynamics does not automatically provide a straightforward answer to the “what caused what?” question. Counterfactual accounts of actual causation, based on graphical models paired with system interventions, have demonstrated initial success in addressing specific problem cases, in line with intuitive causal judgments. Here, we start from a set of basic requirements for causation (realization, composition, information, integration, and exclusion) and develop a rigorous, quantitative account of actual causation, that is generally applicable to discrete dynamical systems. We present a formal framework to evaluate these causal requirements based on system interventions and partitions, which considers all counterfactuals of a state transition. This framework is used to provide a complete causal account of the transition by identifying and quantifying the strength of all actual causes and effects linking the two consecutive system states. Finally, we examine several exemplary cases and paradoxes of causation and show that they can be illuminated by the proposed framework for quantifying actual causation.

## 1. Introduction

The nature of cause and effect has been much debated in both philosophy and the sciences. To date, there is no single widely-accepted account of causation, and the various sciences focus on different aspects of the issue [1]. In physics, no formal notion of causation seems to even be required for describing the dynamical evolution of a system by a set of mathematical equations. At most, the notion of causation is reduced to the basic requirement that causes must precede and be able to influence their effects—no further constraints are imposed with regard to “what caused what”.

However, a detailed record of “what happened” prior to a particular occurrence rarely provides a satisfactory explanation for *why* it occurred in causal, mechanistic terms (see Theory 2.2 for a formal definition of the term “occurrence” as a set of random variables in a particular state at a particular time). As an example, take AlphaGo, the deep neural network that repeatedly defeated human champions in the game Go [2]. Understanding why AlphaGo chose a particular move is a non-trivial problem [3], even though all its network parameters and its state evolution can be recorded in detail. Identifying “what caused what” becomes particularly difficult in complex systems with a distributed, recurrent architecture and wide-ranging interactions, as is typical for biological (neural) networks, including the brain [4,5].

Our interest, here, lies in the principled analysis of *actual causation* in discrete distributed dynamical systems, such as artificial neural networks, computers made of logic gates, or cellular automata, but also simple models of biological brain circuits or gene regulatory networks. In contrast with *general* (or *type*) *causation*, which addresses the question of whether the type of occurrence *A* generally “brings about” the type of occurrence *B*, the underlying notion of *actual* (or *token*) *causation* addresses the question of “what caused what”, given a specific occurrence *A* followed by a specific occurrence *B*. For example, what part of the particular pattern on the board caused AlphaGo to decide on this particular move? As highlighted by the AlphaGo example, even with detailed knowledge of all circumstances, the prior system state, and the outcome, there often is no straightforward answer to the “what caused what” question. This has also been demonstrated by a long list of controversial examples conceived, analyzed, and debated primarily by philosophers (e.g., [6,7,8,9,10,11,12]).

A number of attempts to operationalize the notion of causation and to give it a formal description have been developed, most notably in computer science, probability theory, statistics [7,13,14,15,16], the law [17], and neuroscience, (e.g., [18]). Graphical methods paired with system interventions [7] have proven to be especially valuable for developing causal explanations. Given a causal network that represents how the state of each variable depends on other system variables by a “structural equation” [7], it is possible to evaluate the effects of interventions imposed from outside the network by setting certain variables to a specific value. This operation has been formalized by Pearl, who introduced the “do-operator”, do(X=x), which signifies that a subset of system variables *X* has been actively set into state *x*, rather than being passively observed in this state [7]. As statistical dependence does not imply causal dependence, the conditional probability of occurrence *B* after observing occurrence *A*, p(B∣A), may differ from the probability of occurrence *B* after enforcing *A*, p(B∣do(A)). Causal networks are a specific subset of “Bayesian” networks, that explicitly represent *causal* dependencies consistent with interventional probabilities.

The causal network approach has also been applied to the case of *actual causation* [7,8,11,19,20,21], where system interventions can be used to evaluate whether (and to what extent) an occurrence was necessary or sufficient for a subsequent occurrence by assessing counterfactuals—alternative occurrences “counter to fact” [7,22,23]—within a given causal model. The objective is to define “what it means for *A* to be a cause of *B in a model M*” [12]. Note that counterfactuals, here, strictly refer to the possible states within the system’s state space (other than the actual one) and not to abstract notions, such as other “possible worlds” as in [22] (see also [7], Chapter 7). While promising results have been obtained in specific cases, no single proposal (to date) has characterized actual causation in a universally satisfying manner [10,12]. One concern about existing measures of actual causation is the incremental manner in which they progress; a definition is proposed that satisfies existing examples in the literature, until a new problematic example is discovered, at which point the definition is updated to address the new example [11,24]. While valuable, the problem with such an approach is that one cannot be confident in applying the framework beyond the scope of examples already tested. For example, while these methods are well-explored in simple binary examples, there is less evidence that the methods conform with intuition when we consider the much larger space of non-binary examples. This is especially critical when moving beyond intuitive toy examples to scientific problems where intuition is lacking, such as understanding actual causation in biological or artificial neural networks.

**Figure 1:**
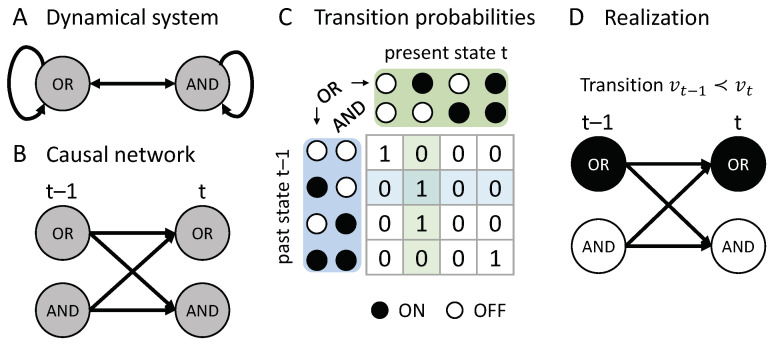
Realization: Dynamical causal network and transition. (**A**) A discrete dynamical system constituted of two interacting elements: An OR- and AND-logic gate, which are updated synchronously at every time step, according to their input-output functions. Arrows denote connections between the elements. (**B**) The same system can be represented as a dynamical causal network over consecutive time steps. (**C**) The system described by its entire set of transition probabilities. As this particular system is deterministic, all transitions have a probability of either p=0 or p=1. (**D**) A realization of a system transient over two time steps, consistent with the system’s transition probabilities: $\left\{(\mathrm{OR},\;{\mathrm{AND})}_{t-1}=10\right\}≺\left\{(\mathrm{OR},\;{\mathrm{AND})}_{t}=10\right\}$.

Our goal is to provide a robust framework for assessing actual causation that is based on general causal principles, and can, thus, be expected to naturally extend beyond simple, binary, and deterministic example cases. Below, we present a formal account of actual causation which is generally applicable to discrete Markovian dynamical systems constituted of interacting elements (see Figure 1). The proposed framework is based on five causal principles identified in the context of integrated information theory (IIT)—namely, existence (here: realization), composition, information, integration, and exclusion [25,26]). Originally developed as a theory of consciousness [27,28], IIT provides the tools to characterize *potential causation*—the causal constraints exerted by a mechanism in a given state.

In particular, our objective is to provide a complete quantitative causal account of “what caused what”, within a transition between consecutive system states. Our approach differs from previous accounts of actual causation in what constitutes a complete causal account: Unlike most accounts of actual causation (e.g., [7,10,12], but see [29]), causal links within a transition are considered from the perspective of *both* causes and effects. Additionally, we not only evaluate actual causes and effects of individual variables, but also actual causes and effects of high-order occurrences, comprising multiple variables. While some existing accounts of actual causation include the notion of being “part of a cause” [12,21], the possibility of multi-variate causes and effects is rarely addressed, or even outright excluded [11].

Despite the differences in what constitutes a complete causal account, our approach remains compatible with the traditional view of actual causation, which considers only actual causes of individual variables (no high-order causation, and no actual effects). In this context, the main difference between our proposed framework and existing “contingency”-based definitions is that we simultaneously consider *all* counterfactual states of the transition, rather than a single contingency (e.g., as in [8,11,19,20,21,30,31]). This allows us to express the causal analysis in probabilistic, informational terms [25,32,33,34], which has the additional benefit that our framework naturally extends from deterministic to probabilistic causal networks, and also from binary to multi-valued variables. Finally, it allows us to quantify the strength of all causal links between occurrences and their causes and effects within the transition.

In the following, we will first formally describe the proposed causal framework of actual causation. We, then, demonstrate its utility on a set of examples, which illustrates the benefits of characterizing both causes and effects, the fact that causation can be compositional, and the importance of identifying irreducible causes and effects for obtaining a complete causal account. Finally, we illustrate several prominent paradoxical cases from the actual causation literature, including overdetermination and prevention, as well as a toy model of an image classifier, based on an artificial neural network.

## 2. Theory

Integrated information theory is concerned with the intrinsic cause-effect power of a physical system (*intrinsic existence*). The IIT formalism [25,27] starts from a discrete distributed dynamical system in its current state and asks how the system elements, alone and in combination (*composition*), constrain the *potential* past and future states of the system (*information*), and whether they do so above and beyond their parts (*integration*). The potential causes and effects of a system subset correspond to the set of elements over which the constraints are maximally informative and integrated (*exclusion*). In the following we aim to translate the IIT account of potential causation into a principled, quantitative framework for *actual* causation, which allows for the evaluation of all actual causes and effects within a state transition of a dynamical system of interacting elements, such as a biological or artificial neural network (see Figure 1). For maximal generality, we will formulate our account of actual causation in the context of dynamical causal networks [32,34,35].

### 2.1. Dynamical Causal Networks

Our starting point is a dynamical causal network: A directed acyclic graph (DAG) $G_{u}=\left(V,E\right)$ with edges *E* that indicate the causal connections among a set of nodes *V* and a given set of background conditions (state of exogenous variables) U=u (see Figure 1B). The nodes in $G_{u}$ represent a set of associated random variables (which we also denote by *V*) with state space $\Omega =\prod_{i}\Omega_{V_{i}}$ and probability function p(v|u),v∈Ω. For any node $V_{i}\in V$, we can define the parents of $V_{i}$ in $G_{u}$ as all nodes with an edge leading into $V_{i}$,

$$pa\left(V_{i}\right)=\left\{V_{j}|e_{ji}\in E\right\}.$$

A causal network $G_{u}$ is dynamical, in the sense that we can define a partition of its nodes *V* into k+1 temporally ordered “slices”, $V=\{V_{0},V_{1},\ldots ,V_{k}\}$, starting with an initial slice without parents ($pa(V_{0})=⌀$) and such that the parents of each successive slice are fully contained within the previous slice ($pa\left(V_{t}\right)\subseteq V_{t-1},\;t=1,\ldots ,k$). This definition is similar to the one proposed in [32], but is stricter, requiring that there are no within-slice causal interactions. This restriction prohibits any “instantaneous causation” between variables (see also [7], Section 1.5) and signifies that $G_{u}$ fulfills the Markov property. Nevertheless, recurrent networks can be represented as dynamical causal models when unfolded in time (see Figure 1B) [20]. The parts of $V=\{V_{0},V_{1},\ldots ,V_{k}\}$ can thus be interpreted as consecutive time steps of a discrete dynamical system of interacting elements (see Figure 1); a particular state V=v, then, corresponds to a system transient over k+1 time steps.

In a *Bayesian* network, the edges of $G_{u}$ fully capture the dependency structure between nodes *V*. That is, for a given set of background conditions, each node is conditionally independent of every other node, given its parents in $G_{u}$, and the probability function can be factored as

$$p\left(v|u\right)=\prod_{i}p\left(v_{i}|pa\left(v_{i}\right),u\right),\;v\in \Omega .$$

For a *causal* network, there is the additional requirement that the edges *E* capture causal dependencies (rather than just correlations) between nodes. This means that the decomposition of p(v∣u) holds, even if the parent variables are actively set into their state as opposed to passively observed in that state (“Causal Markov Condition”, [7,15]),

$$p\left(v|u\right)=\prod_{i}p\left(v_{i}|do\left(pa\left(v_{i}\right),u\right)\right),\;v\in \Omega .$$

As we assume, here, that *U* contains all relevant background variables, any statistical dependencies between $V_{t-1}$ and $V_{t}$ are, in fact, causal dependencies, and cannot be explained by latent external variables (“causal sufficiency”, see [34]). Moreover, because time is explicit in $G_{u}$ and we assume that there is no instantaneous causation, there is no question of the direction of causal influences—it must be that the earlier variables ($V_{t-1}$) influence the later variables ($V_{t}$). By definition, $V_{t-1}$ contains all parents of $V_{t}$ for t=1,…,k. In contrast to the variables *V* within $G_{u}$, the background variables *U* are conditioned to a particular state U=u throughout the causal analysis and are, otherwise, not further considered.

Together, these assumptions imply a transition probability function for *V*, such that the nodes at time *t* are conditionally independent given the state of the nodes at time t−1 (see Figure 1C),

(1) $$\begin{array}{cc} p_{u}\left(v_{t}|v_{t-1}\right) & =p(v_{t}|v_{t-1},u)\\ & =\prod_{i}p\left(v_{i,t}|v_{t-1},u\right)\\ & =\prod_{i}p\left(v_{i,t}|do\left(v_{t-1},u\right)\right),\;\forall \;\left(v_{t-1},v_{t}\right)\in \Omega . \end{array}$$

To reiterate, a dynamical causal network $G_{u}$ describes the causal interactions among a set of nodes (the edges in *E* describe the causal connections between the nodes in *V*) conditional on the state of the background variables *U*, and the transition probability function $p_{u}\left(v_{t}|v_{t-1}\right)$ (Equation (1)) fully captures the nature of these causal dependencies. Note that $p_{u}\left(v_{t}|v_{t-1}\right)$ is generally undefined in the case where $p_{u}\left(v_{t-1}\right)=0$. However, in the present context, it is defined as $p_{u}\left(v_{t}|v_{t-1}\right)=p_{u}\left(v_{t}|do\left(v_{t-1}\right)\right)$ using the $do(v_{t-1})$ operation. The interventional probability $p_{u}\left(v_{t}|do\left(v_{t-1}\right)\right)$ is well-defined for all $v_{t-1}\in \Omega$ and can typically be inferred from the mechanisms associated with the variables in $V_{t}$.

In summary, we assume that $G_{u}$ fully and accurately describes the system of interest for a given set of background conditions. In reality, a causal network reflects assumptions about a system’s elementary mechanisms. Current scientific knowledge must inform which variables to include, what their relevant states are, and how they are related mechanistically [7,36]. Here, we are primarily interested in natural and artificial systems, such as neural networks, for which detailed information about the causal network structure and the mechanisms of individual system elements is often available, or can be obtained through exhaustive experiments. In such systems, counterfactuals can be evaluated by performing experiments or simulations that assess how the system reacts to interventions. The transition probabilities can, in principle, be determined by perturbing the system into all possible states while holding the background variables fixed and observing the resulting transitions. Alternatively, the causal network can be constructed by experimentally identifying the input-output function of each element (i.e., its structural equation [7,34]). Merely observing the system without experimental manipulation is insufficient to identify causal relationships in most situations. Moreover, instantaneous dependencies are frequently observed in (experimentally obtained) time-series data of macroscopic variables, due to unobserved interactions at finer spatio-temporal scales [37]. In this case, a suitable dynamical causal network may still be obtained, simply by discounting such instantaneous dependencies, since these interactions are not due to the macroscopic mechanisms themselves.

Our objective, here, is to formulate a quantitative account of actual causation applicable to any predetermined, dynamical causal network, independent of practical considerations about model selection [12,36]. Confounding issues due to incomplete knowledge, such as estimation biases of probabilities from finite sampling, or latent variables, are, thus, set aside for the present purposes. To what extent and under which conditions the identified actual causes and effects generalize across possible levels of description, or under incomplete knowledge, is an interesting question that we plan to address in future work (see also [38,39]).

### 2.2. Occurrences and Transitions

In general, actual causation can be evaluated over multiple time steps (e.g., considering indirect causal influences). Here, however, we specifically focus on direct causes and effects without intermediary variables or time steps. For this reason, we only consider causal networks containing nodes from two consecutive time points, $V=\{V_{t-1},V_{t}\}$, and define a *transition*, denoted by $v_{t-1}≺v_{t}$, as a realization V=v with $v=(v_{t-1},v_{t})\in \Omega$ and $p_{u}\left(v_{t}|v_{t-1}\right)>0$ (see Figure 1D).

Note that our approach generalizes, in principle, to system transitions across multiple (k>1) time steps, by considering the transition probabilities $p_{u}\left(v_{t}|v_{t-k}\right)$, instead of $p_{u}\left(v_{t}|v_{t-1}\right)$, in Equation (1). While this practice would correctly identify counterfactual dependencies between $v_{t-k}$ and $v_{t}$, it ignores the actual states of the intermediate time steps $\left(v_{t-k+1},\ldots ,v_{t-1}\right)$. As a consequence, this approach cannot, at present, address certain issues regarding causal transitivity across multiple paths, incomplete causal processes in probabilistic causal networks [40], or causal dependencies in non-Markovian systems.

Within a dynamical causal network $G_{u}=\left(V,E\right)$ with $V=\{V_{t-1},V_{t}\}$, our objective is to determine the actual cause or actual effect of occurrences within a transition $v_{t-1}≺v_{t}$. Formally, an *occurrence* is defined to be a sub-state $X_{t-1}=x_{t-1}\subseteq V_{t-1}=v_{t-1}$ or $Y_{t}=y_{t}\subseteq V_{t}=v_{t}$, corresponding to a subset of elements at a particular time and in a particular state. This corresponds to the general usage of the term “event” in the computer science and probability literature. The term “occurrence” was chosen, instead, to avoid philosophical baggage associated with the term “event”.

### 2.3. Cause and Effect Repertoires

**Figure 2:**
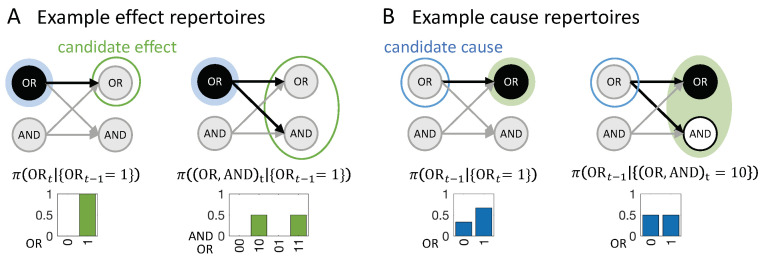
Assessing cause and effect repertoires. (**A**) Example effect repertoires, indicating how the occurrence $\left\{{\mathrm{OR}}_{t-1}=1\right\}$ constrains the future states of ${\mathrm{OR}}_{t}$ (left) and ${\left(\mathrm{OR},\mathrm{AND}\right)}_{t}$ (right) in the causal network shown in Figure 1. (**B**) Example cause repertoires indicating how the occurrences $\left\{{\mathrm{OR}}_{t}=1\right\}$ (left) and $\left\{{\left(\mathrm{OR},\mathrm{AND}\right)}_{t}=10\right\}$ (right) constrain the past states of ${\mathrm{OR}}_{t-1}$. Throughout the manuscript, filled circles denote occurrences, while open circles denote candidate causes and effects. Green shading is used for *t*, blue for t−1. Nodes that are not included in the occurrence or candidate cause/effect are causally marginalized.

Before defining the actual cause or actual effect of an occurrence, we first introduce two definitions from IIT which are useful in characterizing the causal powers of occurrences in a causal network: Cause/effect repertoires and partitioned cause/effect repertoires. In IIT, a cause (or effect) repertoire is a conditional probability distribution that describes how an occurrence (set of elements in a state) constrains the potential past (or future) states of other elements in a system [25,26] (see also [27,41] for a general mathematical definition). In the present context of a transition $v_{t-1}≺v_{t}$, an effect repertoire specifies how an occurrence $x_{t-1}\subseteq v_{t-1}$ constrains the potential future states of a set of nodes $Y_{t}\subseteq V_{t}$. Likewise, a cause repertoire specifies how an occurrence $y_{t}\subseteq v_{t}$ constrains the potential past states of a set of nodes $X_{t-1}\subset V_{t-1}$ (see Figure 2).

The effect and cause repertoire can be derived from the system transition probabilities in Equation (1) by conditioning on the state of the occurrence and *causally marginalizing* the variables outside the occurrence $V_{t-1}\backslash X_{t-1}$ and $V_{t}\backslash Y_{t}$ (see Discussion 4.1). Causal marginalization serves to remove any contributions to the repertoire from variables outside the occurrence by averaging over all their possible states. Explicitly, for a single node $Y_{i,t}$, the effect repertoire is:

(2) $$\pi \left(Y_{i,t}|x_{t-1}\right)=\frac{1}{|\Omega_{W}|}\sum_{w\in \Omega_{W}}p_{u}\left(Y_{i,t}|\mathrm{do}\left(x_{t-1},W=w\right)\right),$$

where $W=V_{t-1}\backslash X_{t-1}$ with state space $\Omega_{W}$. Note that, for causal marginalization, each possible state $W=w\in \Omega_{W}$ is given the same weight $|\Omega_{W}{|}^{-1}$ in the average, which corresponds to imposing a uniform distribution over all $w\in \Omega_{W}$. This ensures that the repertoire captures the constraints due to the occurrence, and not to whatever external factors might bias the variables in *W* to one state or another (this is discussed in more detail in Section 4.1).

In graphical terms, causal marginalizing implies that the connections from all $W_{i}\in W$ to $Y_{i,t}$ are “cut” and independently replaced by an un-biased average across the states of the respective $W_{i}$, which also removes all dependencies between the variables in *W*. Causal marginalization, thus, corresponds to the notion of cutting edges proposed in [34]. However, instead of feeding all open ends with the product of the corresponding marginal distributions obtained from the observed joint distribution, as in Equation (7) of [34], here we impose a uniform distribution $p=\frac{1}{|\Omega_{W}|},\forall w\in \Omega_{W}$, as we are interested in quantifying mechanistic dependencies, which should not depend on the observed joint distribution.

The complementary cause repertoire of a singleton occurrence $y_{i,t}$, using Bayes’ rule, is:

$$\pi \left(X_{t-1}|y_{i,t}\right)=\sum_{w\in \Omega_{W}}\frac{p_{u}\left(y_{i,t}|\mathrm{do}\left(X_{t-1},W=w\right)\right)}{\sum_{z\in \Omega_{V_{t-1}}}p_{u}\left(y_{i,t}|\mathrm{do}\left(V_{t-1}=z\right)\right)}.$$

In the general case of a multi-variate $Y_{t}$ (or $y_{t}$), the transition probability function $p_{u}\left(Y_{t}|x_{t-1}\right)$ not only contains dependencies of $Y_{t}$ on $x_{t-1}$, but also correlations between the variables in $Y_{t}$ due to common inputs from nodes in $W_{t-1}=V_{t-1}\backslash X_{t-1}$, which should not be counted as constraints due to $x_{t-1}$. To discount such correlations, we define the effect repertoire over a set of variables $Y_{t}$ as the product of the effect repertoires over individual nodes (Equation (2)) (see also [34]):

(3) $$\pi \left(Y_{t}|x_{t-1}\right)=\prod_{i}\pi \left(Y_{i,t}|x_{t-1}\right).$$

In the same manner, we define the cause repertoire of a general occurrence $y_{t}$ over a set of variables $X_{t-1}$ as:

(4) $$\pi \left(X_{t-1}|y_{t}\right)=\frac{\prod_{i}\pi \left(X_{t-1}|y_{i,t}\right)}{\sum_{x\in \Omega_{X_{t-1}}}\prod_{i}\pi \left(X_{t-1}=x|y_{i,t}\right)}.$$

We can also define *unconstrained* cause and effect repertoires, a special case of cause or effect repertoires where the occurrence that we condition on is the empty set. In this case, the repertoire describes the causal constraints on a set of the nodes due to the structure of the causal network, under maximum uncertainty about the states of variables within the network. With the convention that π(⌀)=1, we can derive these unconstrained repertoires directly from the formulas for the cause and effect repertoires, Equations (3) and (4). The unconstrained cause repertoire simplifies to a uniform distribution, representing the fact that the causal network itself imposes no constraint on the possible states of variables in $V_{t-1}$,

(5) $$\pi \left(X_{t-1}\right)={\left|\Omega_{X_{t-1}}\right|}^{-1}.$$

The unconstrained effect repertoire is shaped by the update function of each individual node $Y_{i,t}\in Y_{t}$ under maximum uncertainty about the state of its parents,

(6) $$\pi \left(Y_{t}\right)=\prod_{i}\pi \left(Y_{i,t}\right)=\prod_{i}{\left|\Omega_{W}\right|}^{-1}\sum_{w\in \Omega_{W}}p_{u}\left(Y_{i,t}|\mathrm{do}\left(W=w\right)\right),$$

where $W=V_{t-1}\backslash X_{t-1}=V_{t-1}$, since $X_{t-1}=⌀$.

In summary, the effect and cause repertoires $\pi (Y_{t}|x_{t-1})$ and $\pi (X_{t-1}|y_{t})$, respectively, are conditional probability distributions that specify the causal constraints due to an occurrence on the *potential* past and future states of variables in a causal network $G_{u}$. The cause and effect repertoires discount constraints that are not specific to the occurrence of interest; possible constraints due to the state of variables outside of the occurrence are causally marginalized from the distribution, and constraints due to common inputs from other nodes are avoided by treating each node in the occurrence independently. Thus, we denote cause and effect repertoires with π, to highlight that, in general, $\pi \left(Y_{t}|x_{t-1}\right)\neq p\left(Y_{t}|x_{t-1}\right)$. However, $\pi (Y_{t}|x_{t-1})$ is equivalent to $p(Y_{t}|x_{t-1})$ (the conditional probability imposing a uniform distribution over the marginalized variables), in the special case that all variables $Y_{i,t}\in Y_{t}$ are conditionally independent, given $x_{t-1}$ (see also [34], Remark 1). This is the case, for example, if $X_{t-1}$ already includes all inputs (all parents) of $Y_{t}$, or determines $Y_{t}$ completely.

An objective of IIT is to evaluate whether the causal constraints of an occurrence on a set of nodes are “integrated”, or “irreducible”; that is, whether the individual variables in the occurrence work together to constrain the past or future states of the set of nodes in a way that is not accounted for by the variables taken independently [25,42]. To this end, the occurrence (together with the set of nodes it constrains) is partitioned into independent parts, by rendering the connection between the parts causally ineffective [25,26,34,42]. The *partitioned* cause and effect repertoires describe the residual constraints under the partition. Comparing the partitioned cause and effect repertoires to the intact cause and effect repertoires reveals what is lost or changed by the partition.

**Figure 3:**
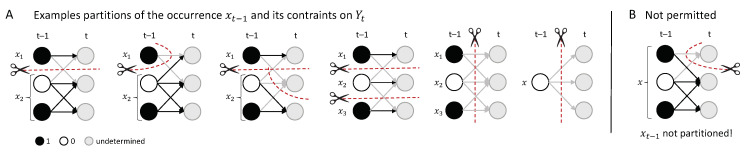
Partitioning the repertoire $\pi (Y_{t}|x_{t-1})$. (**A**) The set of all possible partitions of an occurrence, $\Psi (x_{t-1},Y_{t})$, includes all partitions of $x_{t-1}$ into $2\leq m\leq |x_{t-1}|$ parts, according to Equation (7); as well as the special case $\psi =\{\left(x_{t-1},⌀\right)\}$. Considering this special case a potential partition has the added benefit of allowing us to treat singleton occurrences and multi-variate occurrences in a common framework. (**B**) Except for the special case when the occurrence is completely cut from the nodes it constrains, we generally do not consider cases with m=1 as partitions of the occurrence. The partition must eliminate the possibility of joint constraints of $x_{t-1}$ onto $Y_{t}$. The set of all partitions $\Psi (X_{t-1},y_{t})$ of a cause repertoire $\pi (X_{t-1}|y_{t})$ includes all partitions of $y_{t}$ into $2\leq m\leq |y_{t}|$ parts, according to Equation (9), and, again, the special case of $\psi =\{\left(⌀,y_{t}\right)\}$ for m=1.

A partition ψ of the occurrence $x_{t-1}$ (and the nodes it constrains, $Y_{t}$) into *m* parts is defined as:

(7) $$\psi \left(x_{t-1},Y_{t}\right)=\left\{\left(x_{1,t-1},Y_{1,t}\right),\left(x_{2,t-1},Y_{2,t}\right),\ldots ,\left(x_{m,t-1},Y_{m,t}\right)\right\},$$

such that ${\left\{x_{j,t-1}\right\}}_{j=1}^{m}$ is a partition of $x_{t-1}$ and $Y_{j,t}\subseteq Y_{t}$ with $Y_{j,t}\cap Y_{k,t}=⌀,\;j\neq k$. Note that this includes the possibility that any $Y_{j,t}=⌀$, which may leave a set of nodes $Y_{t}\backslash {⋃}_{j=1}^{m}Y_{j,t}$ completely unconstrained (see Figure 3 for examples and details).

The partitioned effect repertoire of an occurrence $x_{t-1}$ over a set of nodes $Y_{t}$ under a partition ψ is defined as:

(8) $$\pi {\left(Y_{t}|x_{t-1}\right)}_{\psi }=\prod_{j=1}^{m}\pi \left(Y_{j,t}|x_{j,t-1}\right)\times \pi \left(Y_{t}\backslash \overset{m}{\underset{j=1}{⋃}}Y_{j,t}\right).$$

This is the product of the corresponding *m* effect repertoires, multiplied by the unconstrained effect repertoire (Equation (6)) of the remaining set of nodes $Y_{t}\backslash {⋃}_{j=1}^{m}Y_{j,t}$, as these nodes are no longer constrained by any part of $x_{t-1}$ under the partition.

In the same way, a partition ψ of the occurrence $y_{t}$ (and the nodes it constrains $X_{t-1}$) into *m* parts is defined as:

(9) $$\psi \left(X_{t-1},y_{t}\right)=\left\{\left(X_{1,t-1},y_{1,t}\right),\left(X_{2,t-1},y_{2,t}\right),\ldots ,\left(X_{m,t-1},y_{m,t}\right)\right\},$$

such that ${\left\{y_{i,t}\right\}}_{i=1}^{m}$ is a partition of $y_{t}$ and $X_{j,t-1}\subseteq X_{t-1}$ with $X_{j,t-1}\cap X_{k,t-1}=⌀,\;j\neq k$. The partitioned cause repertoire of an occurrence $y_{t}$ over a set of nodes $X_{t-1}$ under a partition ψ is defined as:

(10) $$\pi {\left(X_{t-1}|y_{t}\right)}_{\psi }=\prod_{j=1}^{m}\pi \left(X_{j,t-1}|y_{j,t}\right)\times \pi \left(X_{t-1}\backslash \overset{m}{\underset{j=1}{⋃}}X_{j,t-1}\right),$$

the product of the corresponding *m* cause repertoires multiplied by the unconstrained cause repertoire (Equation (6)) of the remaining set of nodes $X_{t-1}\backslash {⋃}_{j=1}^{m}X_{j,t-1}$, which are no longer constrained by any part of $y_{t}$ due to the partition.

### 2.4. Actual Causes and Actual Effects

The objective of this section is to introduce the notion of a causal account for a transition of interest $v_{t-1}≺v_{t}$ in $G_{u}$ as the set of all causal links between occurrences within the transition. There is a causal link between occurrences $x_{t-1}$ and $y_{t}$ if $y_{t}$ is the actual effect of $x_{t-1}$, or if $x_{t-1}$ is the actual cause of $y_{t}$. Below, we define *causal link*, *actual cause*, *actual effect*, and *causal account*, following five causal principles: Realization, composition, information, integration, and exclusion.

**Realization.** A transition $v_{t-1}≺v_{t}$ must be consistent with the transition probability function of a dynamical causal network $G_{u}$,

$$p_{u}\left(v_{t}|v_{t-1}\right)>0.$$

Only occurrences within a transition $v_{t-1}≺v_{t}$ may have, or be, an actual cause or actual effect (This requirement corresponds to the first clause (“AC1”) of the Halpern and Pearl account of actual causation [20,21]; that is, for C=c to be an actual cause of E=e, both must actually happen in the first place.)

As a first example, we consider the transition $\left\{{\left(\mathrm{OR},\mathrm{AND}\right)}_{t-1}=10\right\}≺\left\{{\left(\mathrm{OR},\mathrm{AND}\right)}_{t}=10\right\}$, shown in Figure 1D. This transition is consistent with the conditional transition probabilities of the system, shown in Figure 1C.

**Figure 4:**
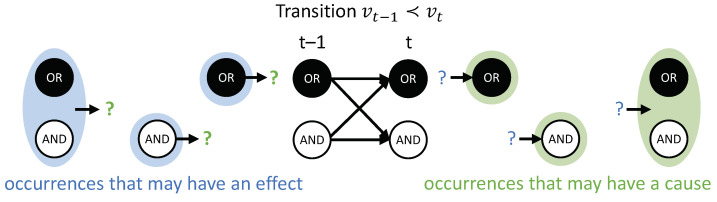
Considering the power set of occurrences. All subsets $x_{t-1}\subseteq v_{t-1}$ and $y_{t}\subseteq v_{t}$ are considered as occurrences which may have an actual effect or an actual cause.

**Figure 5:**
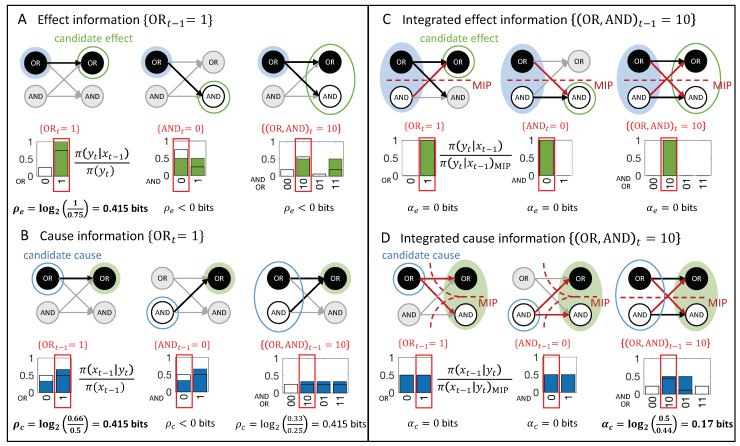
Assessing the cause and effect information, their irreducibility (integration), and the maximum cause/effect (exclusion). (**A**,**B**) Example effect and cause information. The state that actually occurred is selected from the effect or cause repertoire (green is used for effects, blue for causes). Its probability is compared to the probability of the same state when unconstrained (overlaid distributions without fill). All repertoires are based on product probabilities, π (Equations (3) and (4)), that discount correlations due to common inputs when variables are causally marginalized. For example, $\pi (\left\{{\left(\mathrm{OR},\;\mathrm{AND}\right)}_{t}=01\right\})>0$ in (A, right panel), although $p(\left\{{\left(\mathrm{OR},\;\mathrm{AND}\right)}_{t}=01\right\})=0$. (**C**,**D**) Integrated effect and cause information. The probability of the actual state in the effect or cause repertoire is compared against its probability in the partitioned effect or cause repertoire (overlaid distributions without fill). Of all second-order occurrences shown, only $\left\{{\left(\mathrm{OR},\;\mathrm{AND}\right)}_{t}=10\right\}$ irreducibly constrains $\left\{{\left(\mathrm{OR},\;\mathrm{AND}\right)}_{t-1}=10\right\}$. For first-order occurrences, $\alpha_{c/e}=\rho_{c/e}$ (see text). Maximum values are highlighted in bold. If, as in panel (**B**), a superset of a candidate cause or effect specifies the same maximum value, it is excluded by a minimality condition.

**Composition.** Occurrences and their actual causes and effects can be uni- or multi-variate. For a complete causal account of the transition $v_{t-1}≺v_{t}$, *all* causal links between occurrences $x_{t-1}\subseteq v_{t-1}$ and $y_{t}\subseteq v_{t}$ should be considered. For this reason, we evaluate every subset of $x_{t-1}\subseteq v_{t-1}$ as occurrences that may have actual effects and every subset $y_{t}\subseteq v_{t}$ as occurrences that may have actual causes (see Figure 4). For a particular occurrence $x_{t-1}$, all subsets $y_{t}\subseteq v_{t}$ are considered as candidate effects (Figure 5A). For a particular occurrence $y_{t}$, all subsets $x_{t-1}\subseteq v_{t-1}$ are considered as candidate causes (see Figure 5B). In what follows, we refer to occurrences consisting of a single variable as “first-order” occurrences and to multi-variate occurrences as “high-order” occurrences, and, likewise, to “first-order” and “high-order” causes and effects.

In the example transition shown in Figure 4, $\left\{{\mathrm{OR}}_{t-1}=1\right\}$ and $\left\{{\mathrm{AND}}_{t}=0\right\}$ are first-order occurrences that could have an actual effect in $v_{t}$, and $\left\{(\mathrm{OR},\;{\mathrm{AND})}_{t-1}=10\right\}$ is a high-order occurrence that could also have its own actual effect in $v_{t}$. On the other side, $\left\{{\mathrm{OR}}_{t}=1\right\}$, $\left\{{\mathrm{AND}}_{t}=0\right\}$ and $\left\{(\mathrm{OR},\;{\mathrm{AND})}_{t}=10\right\}$ are occurrences (two first-order and one high-order) that could have an actual cause in $v_{t-1}$. To identify the respective actual cause (or effect) of any of these occurrences, we evaluate all possible sets {OR=1}, {AND=0}, and {(OR,AND)=10} at time t−1 (or *t*). Note that, in principle, we also consider the empty set, again using the convention that π(⌀)=1 (see “exclusion”, below).

**Information.** An occurrence must provide information about its actual cause or effect. This means that it should increase the probability of its actual cause or effect compared to its probability if the occurrence is unspecified. To evaluate this, we compare the probability of a candidate effect $y_{t}$ in the effect repertoire of the occurrence $x_{t-1}$ (Equation (3)) to its corresponding probability in the unconstrained repertoire (Equation (6)). In line with information-theoretical principles, we define the effect information $\rho_{e}$ of the occurrence $x_{t-1}$ about a subsequent occurrence $y_{t}$ (the candidate effect) as:

(11) $$\rho_{e}\left(x_{t-1},y_{t}\right)=\log_{2}\left(\frac{\pi (y_{t}|x_{t-1})}{\pi (y_{t})}\right).$$

In words, the effect information $\rho_{e}$ is the relative increase in probability of an occurrence at *t* when constrained by an occurrence at t−1, compared to when it is unconstrained. A positive effect information $\rho_{e}\left(x_{t-1},y_{t}\right)>0$ means that the occurrence $x_{t-1}$ makes a positive difference in bringing about $y_{t}$. Similarly, we compare the probability of a candidate cause $x_{t-1}$ in the cause repertoire of the occurrence $y_{t}$ (Equation (4)) to its corresponding probability in the unconstrained repertoire (Equation (5)). Thus, we define the cause information $\rho_{c}$ of the occurrence $y_{t}$ about a prior occurrence $x_{t-1}$ (the candidate cause) as:

(12) $$\rho_{c}\left(x_{t-1},y_{t}\right)=\log_{2}\left(\frac{\pi (x_{t-1}|y_{t})}{\pi (x_{t-1})}\right).$$

In words, the cause information $\rho_{c}$ is the relative increase in probability of an occurrence at t−1 when constrained by an occurrence at *t*, compared to when it is unconstrained. Note that the unconstrained repertoire (Equations (5) and (6)) is an average over all possible states of the occurrence. The cause and effect information thus take all possible counterfactual states of the occurrence into account in determining the strength of constraints.

In an information-theoretic context, the formula $\log_{2}\left(p(x|y)/p(x)\right)$ is also known as the “pointwise mutual information” (see [43], Chapter 2). While the pointwise mutual information is symmetric, the cause and effect information of an occurrence pair $\left(x_{t-1},y_{t}\right)$ are not always identical, as they are defined based on the product probabilities in Equations (3) and (4). Nevertheless, $\rho_{e}$ and $\rho_{c}$ can be interpreted as the number of bits of information that one occurrence specifies about the other.

In addition to the mutual information, $\rho_{e/c}\left(x_{t-1},y_{t}\right)$ is also related to information-theoretic divergences that measure differences in probability distributions, such as the Kullback–Leibler divergence $D_{KL}\left(p(x|y)||p(x)\right)$, which corresponds to an average of $\log_{2}\left(p(x|y)/p(x)\right)$ over all states $x\in \Omega_{X}$, weighted by p(x∣y). Here, we do not include any such weighting factor, since the transition specifies which states actually occurred. While other definitions of cause and effect information are, in principle, conceivable, $\rho_{e/c}\left(x_{t-1},y_{t}\right)$ captures the notion of information in a general sense and in basic terms.

Note that $\rho_{e}>0$ is a necessary, but not sufficient, condition for $y_{t}$ to be an actual effect of $x_{t-1}$ and $\rho_{c}>0$ is a necessary, but not sufficient, condition for $x_{t-1}$ to be an actual cause of $y_{t}$. Further, $\rho_{c/e}=0$ if and only if conditioning on the occurrence does not change the probability of a potential cause or effect, which is always the case when conditioning on the empty set.

Occurrences $x_{t-1}$ that lower the probability of a subsequent occurrence $y_{t}$ have been termed “preventative causes” by some [33]. Rather than counting a negative effect information $\rho_{e}\left(x_{t-1},y_{t}\right)<0$ as indicating a possible “preventative effect”, we take the stance that such an occurrence $x_{t-1}$ has no effect on $y_{t}$, since it actually predicts other occurrences $Y_{t}=\neg y_{t}$ that did not happen. By the same logic, a negative cause information $\rho_{c}\left(x_{t-1},y_{t}\right)<0$ means that $x_{t-1}$ is not a cause of $y_{t}$ within the transition. Nevertheless, the current framework can, in principle, quantify the strength of possible “preventative” causes and effects.

In Figure 5A, the occurrence $\left\{{\mathrm{OR}}_{t-1}=1\right\}$ raises the probability of $\left\{{\mathrm{OR}}_{t}=1\right\}$, and vice versa (Figure 5B), with $\rho_{e}\left(\left\{{\mathrm{OR}}_{t-1}=1\right\},\left\{{\mathrm{OR}}_{t}=1\right\}\right)=\rho_{c}\left(\left\{{\mathrm{OR}}_{t}=1\right\},\left\{{\mathrm{OR}}_{t-1}=1\right\}\right)=0.415$ bits. By contrast, the occurrence $\left\{{\mathrm{OR}}_{t-1}=1\right\}$ lowers the probability of occurrence $\left\{{\mathrm{AND}}_{t}=0\right\}$ and also of the second-order occurrence $\left\{(\mathrm{OR},\;{\mathrm{AND})}_{t}=10\right\}$, compared to their unconstrained probabilities. Thus, neither $\left\{{\mathrm{AND}}_{t}=0\right\}$ nor $\left\{(\mathrm{OR},\;{\mathrm{AND})}_{t}=10\right\}$ can be actual effects of $\left\{{\mathrm{OR}}_{t-1}=1\right\}$. Likewise, the occurrence $\left\{{\mathrm{OR}}_{t}=1\right\}$ lowers the probability of $\left\{{\mathrm{AND}}_{t-1}=0\right\}$, which can, thus, not be its actual cause.

**Integration.** A high-order occurrence must specify more information about its actual cause or effect than its parts when they are considered independently. This means that the high-order occurrence must increase the probability of its actual cause or effect beyond the value specified by its parts.

As outlined in Section 2.3, a partitioned cause or effect repertoire specifies the residual constraints of an occurrence after applying a partition ψ. We quantify the amount of information specified by the parts of an occurrence based on partitioned cause/effect repertoires (Equations (8) and (10)). We define the effect information under a partition ψ as

(13) $$\rho_{e}{\left(x_{t-1},y_{t}\right)}_{\psi }=\log_{2}\left(\frac{\pi {\left(y_{t}|x_{t-1}\right)}_{\psi }}{\pi (y_{t})}\right),$$

and the cause information under a partition ψ as

(14) $$\rho_{c}{\left(x_{t-1},y_{t}\right)}_{\psi }=\log_{2}\left(\frac{\pi {\left(x_{t-1}|y_{t}\right)}_{\psi }}{\pi (x_{t-1})}\right).$$

The information a high-order occurrence specifies about its actual cause or effect is integrated to the extent that it exceeds the information specified under *any* partition ψ. Out of all permissible partitions $\Psi (x_{t-1},Y_{t})$ (Equation (7)), or $\Psi (X_{t-1},y_{t})$ (Equation (9)), the partition that reduces the effect or cause information the least is denoted the “minimum information partition” (MIP) [25,26], respectively:

$$\mathrm{MIP}=\underset{\psi \in \Psi (x_{t-1},Y_{t})}{arg min}\left(\rho_{e}\left(x_{t-1},y_{t}\right)-\rho_{e}{\left(x_{t-1},y_{t}\right)}_{\psi }\right)$$

or

$$\mathrm{MIP}=\underset{\psi \in \Psi (X_{t-1},y_{t})}{arg min}\left(\rho_{c}\left(x_{t-1},y_{t}\right)-\rho_{c}{\left(x_{t-1},y_{t}\right)}_{\psi }\right).$$

We can, then, define the integrated effect information $\alpha_{e}$ as the difference between the effect information and the information under the MIP:

(15) $$\alpha_{e}\left(x_{t-1},y_{t}\right)=\rho_{e}\left(x_{t-1},y_{t}\right)-\rho_{e}{\left(x_{t-1},y_{t}\right)}_{\mathrm{MIP}}=\log_{2}\left(\frac{\pi (y_{t}|x_{t-1})}{\pi {\left(y_{t}|x_{t-1}\right)}_{\mathrm{MIP}}}\right),$$

and the integrated cause information $\alpha_{c}$ as:

(16) $$\alpha_{c}\left(x_{t-1},y_{t}\right)=\rho_{c}\left(x_{t-1},y_{t}\right)-\rho_{c}{\left(x_{t-1},y_{t}\right)}_{\mathrm{MIP}}=\log_{2}\left(\frac{\pi (x_{t-1}|y_{t})}{\pi {\left(x_{t-1}|y_{t}\right)}_{\mathrm{MIP}}}\right).$$

For first-order occurrences $x_{i,t-1}$ or $y_{i,t-1}$, there is only one way to partition the occurrence ($\psi =\{\left(x_{i,t-1},⌀\right)\}$ or $\psi =\{\left(y_{i,t},⌀\right)\}$), which is necessarily the MIP, leading to $\alpha_{e}\left(x_{i,t-1},y_{t}\right)=\rho_{e}\left(x_{i,t-1},y_{t}\right)$ or $\alpha_{c}\left(x_{t-1},y_{i,t}\right)=\rho_{c}\left(x_{t-1},y_{i,t}\right)$, respectively.

A positive integrated effect information ($\alpha_{e}\left(x_{t-1},y_{t}\right)>0$) signifies that the occurrence $x_{t-1}$ has an irreducible effect on $y_{t}$, which is necessary, but not sufficient, for $y_{t}$ to be an actual effect of $x_{t-1}$. Likewise, a positive integrated cause information ($\alpha_{c}\left(x_{t-1},y_{t}\right)>0$) means that $y_{t}$ has an irreducible cause in $x_{t-1}$, which is a necessary, but not sufficient, condition for $x_{t-1}$ to be an actual cause of $y_{t}$.

In our example transition, the occurrence $\left\{(\mathrm{OR},\;{\mathrm{AND})}_{t-1}=10\right\}$ (Figure 5C) is reducible. This is because $\left\{{\mathrm{OR}}_{t-1}=1\right\}$ is sufficient to determine that $\left\{{\mathrm{OR}}_{t}=1\right\}$ with probability 1 and $\left\{{\mathrm{AND}}_{t-1}=0\right\}$ is sufficient to determine that {AND=0} with probability 1. Thus, there is nothing to be gained by considering the two nodes together as a second-order occurrence. By contrast, the occurrence $\left\{(\mathrm{OR},\;{\mathrm{AND})}_{t}=10\right\}$ determines the particular past state $\left\{(\mathrm{OR},\;{\mathrm{AND})}_{t-1}=10\right\}$ with higher probability than the two first-order occurrences $\left\{{\mathrm{OR}}_{t}=1\right\}$ and $\left\{{\mathrm{AND}}_{t}=0\right\}$, taken separately (Figure 5D, right). Thus, the second-order occurrence $\left\{(\mathrm{OR},\;{\mathrm{AND})}_{t}=10\right\}$ is irreducible over the candidate cause $\left\{(\mathrm{OR},\;{\mathrm{AND})}_{t-1}=10\right\}$ with $\alpha_{c}\left(\left\{(\mathrm{OR},\;{\mathrm{AND})}_{t-1}=10\right\},\left\{(\mathrm{OR},\;{\mathrm{AND})}_{t}=10\right\}\right)=0.17$ bits (see Discussion 4.4).

**Exclusion:** An occurrence should have at most one actual cause and one actual effect (which, however, can be multi-variate; that is, a high-order occurrence). In other words, only one occurrence $y_{t}\subseteq v_{t}$ can be the actual effect of an occurrence $x_{t-1}$, and only one occurrence $x_{t-1}\subseteq v_{t-1}$ can be the actual cause of an occurrence $y_{t}$.

It is possible that there are multiple occurrences $y_{t}\subseteq v_{t}$ over which $x_{t-1}$ is irreducible ($\alpha_{e}\left(x_{t-1},y_{t}\right)>0$), as well as multiple occurrences $x_{t-1}\subseteq v_{t-1}$ over which $y_{t}$ is irreducible ($\alpha_{c}\left(x_{t-1},y_{t}\right)>0$). The integrated effect or cause information of an occurrence quantifies the strength of its causal constraint on a candidate effect or cause. When there are multiple candidate causes or effects for which $\alpha_{c/e}\left(x_{t-1},y_{t}\right)>0$, we select the strongest of those constraints as its actual cause or effect (that is, the one that maximizes α). Note that adding unconstrained variables to a candidate cause (or effect) does not change the value of α, as the occurrence still specifies the same irreducible constraints about the state of the extended candidate cause (or effect). For this reason, we include a “minimality” condition, such that no subset of an actual cause or effect should have the same integrated cause or effect information. This minimality condition between overlapping candidate causes or effects is related to the third clause (“AC3”) in the various Halpern–Pearl (HP) accounts of actual causation [20,21], which states that no subset of an actual cause should also satisfy the conditions for being an actual cause. Under uncertainty about the causal model, or other practical considerations, the minimality condition could, in principle, be replaced by a more elaborate criterion, similar to, for example, the Akaike information criterion (AIC) that weighs increases in causal strength, as measured here, against the number of variables included in the candidate cause or effect.

We define the irreducibility of an occurrence as its maximum integrated effect (or cause) information over all candidate effects (or causes),

$$\alpha_{e}^{\max}\left(x_{t-1}\right)=\max_{y_{t}\subseteq v_{t}}\alpha_{e}\left(x_{t-1},y_{t}\right),$$

and

$$\alpha_{c}^{\max}\left(y_{t}\right)=\max_{x_{t-1}\subseteq v_{t-1}}\alpha_{c}\left(x_{t-1},y_{t}\right).$$

Considering the empty set as a possible cause or effect guarantees that the minimal value that $\alpha^{\max}$ can take is 0. Accordingly, if $\alpha^{\max}=0$, then the occurrence is said to be reducible, and it has is no actual cause or effect.

For the example in Figure 2A, $\left\{{\mathrm{OR}}_{t}=1\right\}$ has two candidate causes with $\alpha_{c}^{\max}\left(\left\{{\mathrm{OR}}_{t}=1\right\}\right)=0.415$ bits, the first-order occurrence $\left\{{\mathrm{OR}}_{t-1}=1\right\}$ and the second-order occurrence $\left\{(\mathrm{OR},\;{\mathrm{AND})}_{t-1}=10\right\}$. In this case, $\left\{{\mathrm{OR}}_{t-1}=1\right\}$ is the actual cause of $\left\{{\mathrm{OR}}_{t}=1\right\}$, by the minimality condition across overlapping candidate causes.

The exclusion principle avoids causal over-determination, which arises from counting multiple causes or effects for a single occurrence. Note, however, that symmetries in $G_{u}$ can give rise to genuine indeterminism about the actual cause or effect (see Results 3). This is the case if multiple candidate causes (or effects) are maximally irreducible and they are not simple sub- or super-sets of each other. Upholding the causal exclusion principle, such degenerate cases are resolved by stipulating that the *one* actual cause remains undetermined between all minimal candidate causes (or effects).

To summarize, we formally translate the five causal principles of IIT into the following requirements for actual causation:
**Realization:**There is a dynamical causal network $G_{u}$ and a transition $v_{t-1}≺v_{t}$, such that $p_{u}\left(v_{t}|v_{t-1}\right)>0$.**Composition:**All $x_{t-1}\subseteq v_{t-1}$ may have actual effects and be actual causes, and all $y_{t}\subseteq v_{t}$ may have actual causes and be actual effects.
**Information:**Occurrences must increase the probability of their causes or effects ($\rho (x_{t-1},y_{t})>0$).**Integration:**Moreover, they must do so above and beyond their parts ($\alpha (x_{t-1},y_{t})>0$).**Exclusion:**An occurrence has only one actual cause (or effect), and it is the occurrence that maximizes $\alpha_{c}$ (or $\alpha_{e}$).

Having established the above causal principles, we now formally define the actual cause and the actual effect of an occurrence within a transition $v_{t-1}≺v_{t}$ of the dynamical causal network $G_{u}$:

**Definition** **1.** *Within a transition $v_{t-1}≺v_{t}$ of a dynamical causal network $G_{u}$, the actual cause of an occurrence $y_{t}\subseteq v_{t}$ is an occurrence $x_{t-1}\subseteq v_{t-1}$ which satisfies the following conditions:*

*1.*  *The integrated cause information of $y_{t}$ over $x_{t-1}$ is maximal*
  $$\alpha_{c}\left(x_{t-1},y_{t}\right)=\alpha^{\max}\left(y_{t}\right);\;\mathrm{and}$$
*2.*  *No subset of $x_{t-1}$ satisfies condition (1)*
  $$\alpha_{c}\left(x_{t-1}^{\prime },y_{t}\right)=\alpha^{\max}\left(y_{t}\right)\Rightarrow x_{t-1}^{\prime }⊄x_{t-1}.$$

Define the set of all occurrences that satisfy the above conditions as $x^{*}\left(y_{t}\right)$. As an occurrence can have, at most, one actual cause, there are three potential outcomes:

*1.*  If $x^{*}\left(y_{t}\right)=\left\{x_{t-1}\right\}$, then $x_{t-1}$ is the actual cause of $y_{t}$;
*2.*  if $|x^{*}\left(y_{t}\right)|>1$ then the actual cause of $y_{t}$ is indeterminate; and
*3.*  if $x^{*}\left(y_{t}\right)=\left\{⌀\right\}$, then $y_{t}$ has no actual cause.

**Definition** **2.** *Within a transition $v_{t-1}≺v_{t}$ of a dynamical causal network $G_{u}$, the actual effect of an occurrence $x_{t-1}\subseteq v_{t-1}$ is an occurrence $y_{t}\subseteq v_{t}$ which satisfies the following conditions:*

*1.*  *The integrated effect information of $x_{t-1}$ over $y_{t}$ is maximal*
  $$\alpha_{e}\left(x_{t-1},y_{t}\right)=\alpha^{\max}\left(x_{t-1}\right);\;\mathrm{and}$$
*2.*  *No subset of $y_{t}$ satisfies condition (1)*
  $$\alpha_{e}\left(x_{t-1},y_{t}^{\prime }\right)=\alpha^{\max}\left(x_{t-1}\right)\Rightarrow y_{t}^{\prime }⊄y_{t}.$$

Define the set of all occurrences that satisfy the above conditions as $y^{*}\left(x_{t-1}\right)$. As an occurrence can have, at most, one actual effect, there are three potential outcomes:

*1.*  If $y^{*}\left(x_{t-1}\right)=\left\{y_{t}\right\}$, then $y_{t}$ is the actual effect of $x_{t-1}$;
*2.*  if $|y^{*}\left(x_{t-1}\right)|>1$ then the actual effect of $x_{t-1}$ is indeterminate; and
*3.*  if $y^{*}\left(x_{t-1}\right)=\left\{⌀\right\}$, then $x_{t-1}$ has no actual effect.

Based on Definitions 1 and 2:

**Definition** **3.** Within a transition $v_{t-1}≺v_{t}$ of a dynamical causal network $G_{u}$, a causal link is an occurrence $x_{t-1}\subseteq v_{t-1}$ with $\alpha_{e}^{\max}\left(x_{t-1}\right)>0$ and actual effect $y^{*}\left(x_{t-1}\right)$,

$$x_{t-1}\to y^{*}\left(x_{t-1}\right),$$

or an occurrence $y_{t}\subseteq v_{t}$ with $\alpha_{c}^{\max}\left(y_{t}\right)>0$ and actual cause $x^{*}\left(y_{t}\right)$,

$$x^{*}\left(y_{t}\right)\leftarrow y_{t}.$$

An integrated occurrence defines a single causal link, regardless of whether the actual cause (or effect) is unique or indeterminate. When the actual cause (or effect) is unique, we sometimes refer to the actual cause (or effect) explicitly in the causal link, $x_{t-1}\leftarrow y_{t}$ (or $x_{t-1}\to y_{t}$). The *strength* of a causal link is determined by its $\alpha_{e}^{\max}$ or $\alpha_{c}^{\max}$ value. Reducible occurrences ($\alpha^{\max}=0$) cannot form a causal link.

**Definition** **4.** For a transition $v_{t-1}≺v_{t}$ of a dynamical causal network $G_{u}$, the causal account, $C(v_{t-1}≺v_{t})$, is the set of all causal links $x_{t-1}\to y^{*}\left(x_{t-1}\right)$ and $x^{*}\left(y_{t}\right)\leftarrow y_{t}$ within the transition.

Under this definition, all actual causes and actual effects contribute to the causal account $C(v_{t-1}≺v_{t})$. Notably, the fact that there is a causal link $x_{t-1}\to y_{t}$ does not necessarily imply that the reverse causal link $x_{t-1}\leftarrow y_{t}$ is also present, and vice versa. In other words, just because $y_{t}$ is the actual effect of $x_{t-1}$, the occurrence $x_{t-1}$ does not have to be the actual cause of $y_{t}$. It is, therefore, not redundant to include both directions in $C(v_{t-1}≺v_{t})$, as illustrated by the examples of over-determination and prevention in the Results section (see, also, Discussion 4.2).

**Figure 6:**
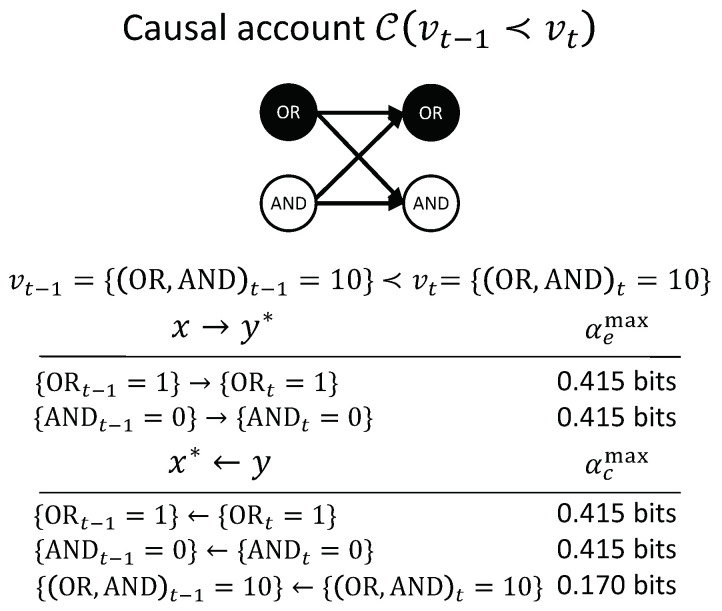
Causal Account. There are two first-order occurrences with actual effects and actual causes. In addition, the second-order occurrence $\left\{(\mathrm{OR},\;{\mathrm{AND})}_{t}=10\right\}$ has an actual cause $\left\{(\mathrm{OR},\;{\mathrm{AND})}_{t-1}=10\right\}$.

Figure 6 shows the entire causal account of our example transition. Intuitively, in this simple example, $\left\{{\mathrm{OR}}_{t-1}=1\right\}$ has the actual effect $\left\{{\mathrm{OR}}_{t}=1\right\}$ and is also the actual cause of $\left\{{\mathrm{OR}}_{t}=1\right\}$, and the same for $\left\{{\mathrm{AND}}_{t-1}=0\right\}$ and {AND=0}. Nevertheless, there is also a causal link between the second-order occurrence $\left\{(\mathrm{OR},\;{\mathrm{AND})}_{t}=10\right\}$ and its actual cause $\left\{(\mathrm{OR},\;{\mathrm{AND})}_{t-1}=10\right\}$, which is irreducible to its parts, as shown in Figure 5D (right). However, there is no complementary link from $\left\{(\mathrm{OR},\;{\mathrm{AND})}_{t}=10\right\}$ to $\left\{(\mathrm{OR},\;{\mathrm{AND})}_{t-1}=10\right\}$, as it is reducible (Figure 5C, right). The causal account, shown in Figure 6, provides a complete causal explanation for “what happened” and “what caused what” in the transition $\left\{(\mathrm{OR},\;{\mathrm{AND})}_{t-1}=10\right\}≺\left\{(\mathrm{OR},\;{\mathrm{AND})}_{t}\;=\;10\right\}$.

Similar to the notion of system-level integration in IIT [25,26], the principle of integration can also be applied to the causal account as a whole, not only to individual causal links (see Appendix A). In this way, it is possible to evaluate to what extent the transition $v_{t-1}≺v_{t}$ is irreducible to its parts, which is quantified by $A(v_{t-1}≺v_{t})$.

In summary, the measures defined in this section provide the means to exhaustively assess “what caused what” in a transition $v_{t-1}≺v_{t}$, and to evaluate the strength of specific causal links of interest under a particular set of background conditions, U=u.

Software to analyze transitions in dynamical causal networks with binary variables is freely available within the “PyPhi” toolbox for integrated information theory [44] at https://github.com/wmayner/pyphi, including documentation at https://pyphi.readthedocs.io/en/stable/examples/actual_causation.html.

## 3. Results

In the following, we will present a series of examples to illustrate the quantities and objects defined in the theory section and address several dilemmas taken from the literature on actual causation. While indeterminism may play a fundamental role in physical causal models, the existing literature on actual causation largely focuses on deterministic problem cases. For ease of comparison, most causal networks analyzed in the following are, thus, deterministic, corresponding to prominent test cases of counterfactual accounts of actual causation (e.g., [8,11,19,20,21,45]).

### 3.1. Same Transition, Different Mechanism: Disjunction, Conjunction, Bi-Conditional, and Prevention

**Figure 7:**
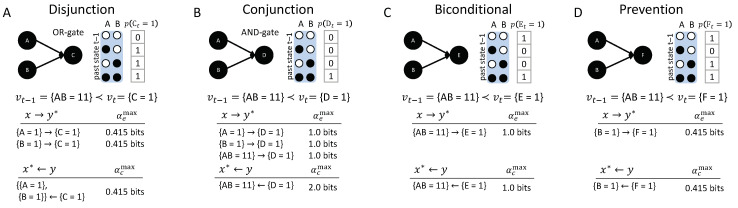
Four dynamically identical transitions can have different causal accounts. Shown are the transitions (top) and their respective causal accounts (bottom).

Figure 7 shows four causal networks of different types of logic gates with two inputs each, all transitioning from the input state $v_{t-1}=\left\{AB=11\right\}$ to the output state $v_{t}=\left\{C=1\right\}$, {D=1}, {E=1}, or {F=1}. From a dynamical point of view, without taking the causal structure of the mechanisms into account, the same occurrences happen in all four situations. However, analyzing the causal accounts of these transitions reveals differences in the number, type, and strength of causal links between occurrences and their actual causes or effects.

**Disjunction:** The first example (Figure 7A, OR-gate), is a case of symmetric over-determination ([7], Chapter 10): each input to *C* would have been sufficient for {C=1}, yet both {A=1} and {B=1} occurred at t−1. In this case, each of the inputs to *C* has an actual effect, {A=1}→{C=1} and {B=1}→{C=1}, as they raise the probability of {C=1} when compared to its unconstrained probability. The high-order occurrence {AB=11}, however, is reducible (with $\alpha_{e}=0$). While both {A=1} and {B=1} have actual effects, by the causal exclusion principle, the occurrence {C=1} can only have one actual cause. As both {A=1}←{C=1} and {B=1}←{C=1} have $\alpha_{c}=\alpha_{c}^{\max}=0.415$ bits, the actual cause of {C=1} is either {A=1} or {B=1}, by Definition 1; which of the two inputs it is remains undetermined, since they are perfectly symmetric in this example. Note that {AB=11}←{C=1} also has $\alpha_{c}=0.415$ bits, but {AB=11} is excluded from being a cause by the minimality condition.

**Conjunction:** In the second example (Figure 7B, AND-gate), both {A=1} and {B=1} are necessary for {D=1}. In this case, each input alone has an actual effect, {A=1}→{C=1} and {B=1}→{C=1} (with higher strength than in the disjunctive case); here, also, the second-order occurrence of both inputs together has an actual effect, {AB=11}→{D=1}. Thus, there is a composition of actual effects. Again, the occurrence {D=1} can only have one actual cause; here, it is the second-order cause {AB=11}, the only occurrence that satisfies the conditions in Definition 1 with $\alpha_{c}=\alpha_{c}^{\max}=2.0$.

The two examples in Figure 7A,B are often referred to as the disjunctive and conjunctive versions of the “forest-fire” example [12,20,21], where lightning and/or a match being dropped result in a forest fire. In the case that lightning strikes and the match is dropped, {A=1} and {B=1} are typically considered two separate (first-order) causes in both the disjunctive and conjunctive version (e.g., [20]). This result is not a valid solution within our proposed account of actual causation, as it violates the causal exclusion principle. We explicitly evaluate the high-order occurrence {AB=11} as a candidate cause, in addition to {A=1} and {B=1}. In line with the distinct logic structure of the two examples, we identify the high-order occurrence {AB=11} as the actual cause of {D=1} in the conjunctive case, while we identify either {A=1} or {B=1} as the actual cause of {C=1} in the disjunctive case, but not both. By separating actual causes from actual effects, acknowledging causal composition, and respecting the causal exclusion principle, our proposed causal analysis can illuminate and distinguish all situations displayed in Figure 7.

**Bi-conditional**: The significance of high-order occurrences is further emphasized by the third example (Figure 7C), where *E* is a “logical bi-conditional” (an XNOR) of its two inputs. In this case, the individual occurrences {A=1} and {B=1} by themselves make no difference in bringing about {E=1}; their effect information is zero. For this reason, they cannot have actual effects and cannot be actual causes. Only the second-order occurrence {AB=11} specifies {E=1}, which is its actual effect {AB=11}→{E=1}. Likewise, {E=1} only specifies the second-order occurrence {AB=11}, which is its actual cause {AB=11}←{E=1}, but not its parts taken separately. Note that the causal strength in this example is lower than in the case of the AND-gate, since, everything else being equal, {D=1} is, mechanistically, a less-likely output than {E=1}.

**Prevention:** In the final example, Figure 7D, all input states but {AB=10} lead to {F=1}. Here, {B=1}→{F=1} and {B=1}←{F=1}, whereas {A=1} does not have an actual effect and is not an actual cause. For this reason, the transition $v_{t-1}≺v_{t}$ is reducible ($A(v_{t-1}≺v_{t})=0$, see Appendix A), since *A* could be partitioned away without loss. This example can be seen as a case of prevention: {B=1} causes {F=1}, which prevents any effect of {A=1}. In a popular narrative accompanying this example, {A=1} is an assassin putting poison in the King’s tea, while a bodyguard administers an antidote {B=1}, and the King survives {F=1} [12]. The bodyguard thus “prevents” the King’s death (However, the causal model is also equivalent to an OR-gate, as can be seen by switching the state labels of *A* from ‘0’ to ‘1’ and vice versa. The discussed transition would correspond to the case of one input to the OR-gate being ‘1’ and the other ‘0’. As the OR-gate switches on (‘1’) in this case, the ‘0’ input has no effect and is not a cause). Note that the causal account is state-dependent: For a different transition, *A* may have an actual effect or contribute to an actual cause; if the bodyguard does not administer the antidote ({B=0}), whether the King survives depends on the assassin (the state of *A*).

Taken together, the above examples demonstrate that the causal account and the causal strength of individual causal links within the account capture differences in sufficiency and necessity of the various occurrences in their respective transitions. Including both actual causes and effects, moreover, contributes to a mechanistic understanding of the transition, since not all occurrences at t−1 with actual effects end up being actual causes of occurrences at *t*.

### 3.2. Linear Threshold Units

A generalization of simple, linear logic gates, such as OR- and AND-gates, are binary linear threshold units (LTUs). Given *n* equivalent inputs $V_{t-1}=\left\{V_{1,t-1},V_{2,t-1},\ldots ,V_{n,t-1}\right\}$ to a single LTU $V_{t}$, $V_{t}$ will turn on (‘1’) if the number of inputs in state ‘1’ exceeds a given threshold *k*,

(17) $$p\left(V_{t}=1|v_{t-1}\right)=\left\{\begin{array}{cc} 1 & \mathrm{if}\;\sum_{i=1}^{n}v_{i,t-1}\geq k,\\ 0 & \mathrm{if}\;\sum_{i=1}^{n}v_{i,t-1}<k. \end{array}\right.$$

LTUs are of great interest, for example, in the field of neural networks, since they comprise one of the simplest model mechanisms for neurons; capturing the notion that a neuron fires if it received sufficient synaptic inputs. One example is a Majority-gate, which outputs ‘1’ if and only if more than half of its inputs are ‘1’.

**Figure 8:**
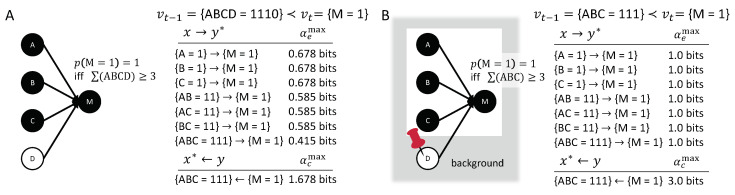
A linear threshold unit with four inputs and threshold k=3 (Majority gate). (**A**) All inputs are considered relevant variables. (**B**) The case D=0 is taken as a fixed background condition (indicated by the red pin).

Figure 8A displays the causal account of a Majority-gate *M* with four inputs for the transition $v_{t-1}=\left\{ABCD=1110\right\}\to v_{t}=\left\{M=1\right\}$. All of the inputs in state ‘1’, as well as their high-order occurrences, have actual effects on {M=1}. Occurrence {D=0}, however, does not work towards bringing about {M=1}: It reduces the probability for {M=1} and, thus, does not contribute to any actual effects or the actual cause. As with the AND-gate in the previous section, there is a composition of actual effects in the causal account. Yet, there is only one actual cause, {ABC=111}←{M=1}. In this case, it happens to be that the third-order occurrence {ABC=111} is minimally sufficient for {M=1}—no smaller set of inputs would suffice. Note, however, that the actual cause is not determined based on sufficiency, but because {ABC=111} is the set of nodes maximally constrained by the occurrence {M=1}. Nevertheless, causal analysis, as illustrated here, will always identify a minimally sufficient set of inputs as the actual cause of an LTU $v_{t}=1$, for any number of inputs *n* and any threshold *k*. Furthermore, any occurrence of input variables $x_{t-1}\subseteq v_{t-1}$ with at most *k* nodes, all in state ‘1’, will be irreducible, with the LTU $v_{t}=1$ as their actual effect.

**Theorem** **1.** *Consider a dynamical causal network $G_{u}$, such that $V_{t}=\left\{Y_{t}\right\}$ is a linear threshold unit with n inputs and threshold k≤n, and $V_{t-1}$ is the set of n inputs to $Y_{t}$. For a transition $v_{t-1}≺v_{t}$, with $y_{t}=1$ and $\sum v_{t-1}\geq k$, the following holds:*

*1.*  *The actual cause of $\left\{Y_{t}=1\right\}$ is an occurrence $\left\{X_{t-1}=x_{t-1}\right\}$ with $|x_{t-1}|=k$ and $\min(x_{t-1})=1$, and*
*2.*  *if $\min(x_{t-1})=1$ and $|x_{t-1}|\leq k$ then the actual effect of $\left\{X_{t-1}=x_{t-1}\right\}$ is $\left\{Y_{t}=1\right\}$; otherwise $\left\{X_{t-1}=x_{t-1}\right\}$ has no actual effect, it is reducible.*

**Proof.**  See Appendix B. □

Note that a LTU in the off (‘0’) state, $\left\{Y_{t}=0\right\}$, has equivalent results with the role of ‘0’ and ‘1’ reversed, and a threshold of n−k. In the case of over-determination (e.g., the transition $v_{t-1}=\left\{ABCD=1111\right\}≺v_{t}=\left\{M=1\right\}$, where all inputs to the Majority-gate are ‘1’), the actual cause will again be a subset of three input nodes in the state ‘1’. However, which of the possible sets remains undetermined, due to symmetry, just as in the case of the OR-gate in Figure 7A.

For comparison, the original and updated Halpern–Pearl (HP) definitions of actual causation [20] generally identify all individual variables in state ’1’ as causes of an LTU $v_{t}=1$. The modified HP definition proposed in [21], roughly speaking, identifies the actual causes as the set of variables whose state needs to be flipped in order to change the outcome, which may vary depending on the state $v_{t-1}$ and the threshold *k*. In the particular example of Figure 8, {A=1}, {B=1}, and {C=1} would count as separate causes. However, in case of the transition $\left\{ABCD=1111\right\}\to v_{t}=\left\{M=1\right\}$, any pair of two inputs would now qualify as a cause of M=1, according to [21].

### 3.3. Distinct Background Conditions

The causal network in Figure 8A considers all inputs to *M* as relevant variables. Under certain circumstance, however, we may want to consider a different set of background conditions. For example, in a voting scenario it may be a given that *D* always votes “no” (D=0). In that case, we may want to analyze the causal account of the transition $v_{t-1}=\left\{ABC=111\right\}≺v_{t}=\left\{M=1\right\}$ in the alternative causal model $G_{u^{\prime }}$, where $\left\{D=0\right\}\in \left\{U^{\prime }=u^{\prime }\right\}$ is treated as a background condition (see Figure 8B). Doing so results in a causal account with the same causal links but higher causal strengths. This captures the intuition that the “yes votes” of *A*, *B*, and *C* are more important if it is already determined that *D* will vote “no”.

The difference between the causal accounts of $v_{t-1}≺v_{t}$ in $G_{u}$, compared to $G_{u^{\prime }}$, moreover, highlights the fact that we explicitly distinguish fixed background conditions U=u from relevant variables *V*, whose counterfactual relations must be considered (see also [46]). While the background variables are fixed in their actual state U=u, all counterfactual states of the relevant variables *V* are considered when evaluating the causal account of $v_{t-1}≺v_{t}$ in $G_{u}$.

### 3.4. Disjunction of Conjunctions

Another case often considered in the actual causation literature is a disjunction of conjunctions (DOC); that is, an OR-operation over two or more AND-operations. In the general case, a disjunction of conjunctions is a variable $V_{t}$ that is a disjunction of *k* conditions, each of which is a conjunction of $n_{j}$ input nodes $V_{t-1}={\left\{{\left\{V_{i,j,t-1}\right\}}_{i=1}^{n_{j}}\right\}}_{j=1}^{k}$,

$$p\left(V_{t}=1|v_{t-1}\right)=\left\{\begin{array}{cc} 0 & \mathrm{if}\;\sum_{i=1}^{n_{j}}v_{i,j,t-1}<n_{j},\;\forall j\\ 1 & \mathrm{otherwise} \end{array}\right..$$

**Figure 9:**
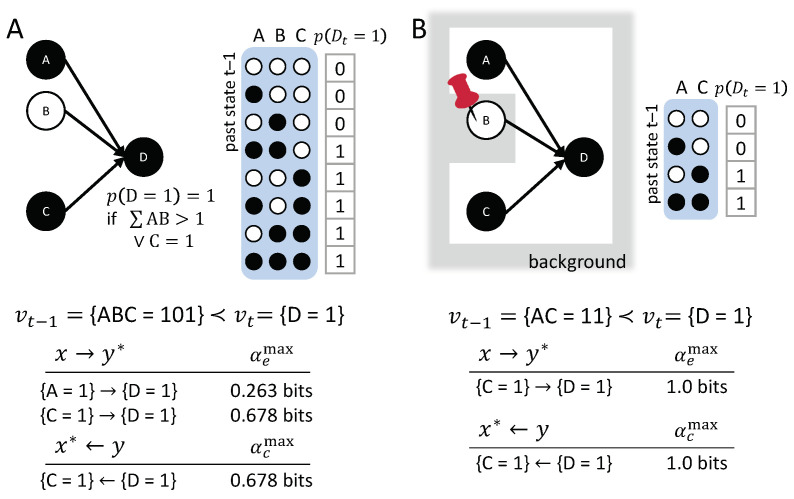
Disjunction of two conjunctions (A∧B)∨C. (**A**) All inputs to *D* are considered relevant variables. (**B**) B=0 is taken as a fixed background condition.

Here, we consider a simple example, (A∧B)∨C (see Figure 9). The debate over this example is mostly concerned with the type of transition shown in Figure 9A: $v_{t-1}=\left\{ABC=101\right\}≺v_{t}=\left\{D=1\right\}$, and the question of whether {A=1} is a cause of {D=1}, even if B=0. One story accompanying this example is: “a prisoner dies either if *A* loads *B*’s gun and *B* shoots, or if *C* loads and shoots his gun, *…*, *A* loads *B*’s gun, *B* does not shoot, but *C* does load and shoot his gun, so that the prisoner dies” [12,47].

The quantitative assessment of actual causes and actual effects can help to resolve issues of actual causation, in this type of example. As shown in Figure 9A, with respect to actual effects, both causal links {A=1}→{D=1} and {C=1}→{D=1} are present, with {C=1} having a stronger actual effect. However, {C=1} is the one actual cause of {D=1}, being the maximally irreducible cause with $\alpha_{c}^{\max}\left(\left\{D=1\right\}\right)=0.678$.

When judging the actual effect of {A=1} at t−1 within the transition $v_{t-1}=\left\{ABC=101\right\}≺v_{t}=\left\{D=1\right\}$, *B* is assumed to be undetermined. By itself, the occurrence {A=1} does raise the probability of occurrence {D=1}, and thus {A=1}→{D=1}.

If we, instead, consider $\left\{B=0\right\}\in \left\{U^{\prime }=u^{\prime }\right\}$ as a fixed background condition and evaluate the transition $v_{t-1}=\left\{AC=11\right\}≺v_{t}=\left\{D=1\right\}$ in $G_{u^{\prime }}$, {A=1} does not have an actual effect anymore (Figure 9B). In this case, the background condition {B=0} prevents {A=1} from having any effect.

The results from this example extend to the general case of disjunctions of conjunctions. In the situation where $v_{t}=1$, the actual cause of $v_{t}$ is a minimally sufficient occurrence. If multiple conjunctive conditions are satisfied, the actual cause of $v_{t}$ remains indeterminate between all minimally sufficient sets (asymmetric over-determination). At t−1, any first-order occurrence in state ‘1’, as well as any high-order occurrence of such nodes that does not overdetermine $v_{t}$, has an actual effect. This includes any occurrence in state all ‘1’ that contains only variables from exactly one conjunction, as well as any high-order occurrence of nodes across conjunctions, which do not fully contain any specific conjunction.

If, instead, $v_{t}=0$, then its actual cause is an occurrence that contains a single node in state ‘0’ from each conjunctive condition. At t−1, any occurrence in state all ‘0’ that does not overdetermine $v_{t}$ has an actual effect, which is any all ‘0’ occurrence that does not contain more than one node from any conjunction.

These results are formalized by the following theorem.

**Theorem** **2.** *Consider a dynamical causal network $G_{u}$, such that $V_{t}=\left\{Y_{t}\right\}$ is a DOC element that is a disjunction of k conditions, each of which is a conjunction of $n_{j}$ inputs, and $V_{t-1}={\left\{{\left\{V_{i,j,t-1}\right\}}_{i=1}^{n_{j}}\right\}}_{j=1}^{k}$ is the set of its $n=\sum_{j}n_{j}$ inputs. For a transition $v_{t-1}≺v_{t}$, the following holds:*

*1.*  *If $y_{t}=1$,*
  *(a)*  *The actual cause of $\left\{Y_{t}=1\right\}$ is an occurrence $\left\{X_{t-1}=x_{t-1}\right\}$ where $x_{t-1}={\left\{x_{i,j,t-1}\right\}}_{i=1}^{n_{j}}\subseteq v_{t-1}$ such that $\min(x_{t-1})=1$; and*
  *(b)*  *the actual effect of $\left\{X_{t-1}=x_{t-1}\right\}$ is $\left\{Y_{t}=1\right\}$ if $\min(x_{t-1})=1$ and $|x_{t-1}|=c_{j}=n_{j}$; otherwise $x_{t-1}$ is reducible.*
*2.*  *If $y_{t}=0$,*
  *(a)*  *The actual cause of $\left\{Y_{t}=0\right\}$ is an occurrence $x_{t-1}\subseteq v_{t-1}$ such that $\max(x_{t-1})=0$ and $c_{j}=1\;\forall \;j$; and*
  *(b)*  *if $\max(x_{t-1})=0$ and $c_{j}\leq 1\;\forall \;j$ then the actual effect of $\left\{X_{t-1}=x_{t-1}\right\}$ is $\left\{Y_{t}=0\right\}$; otherwise $x_{t-1}$ is reducible.*

**Proof.**  See Appendix C. □

### 3.5. Complicated Voting

**Figure 10:**
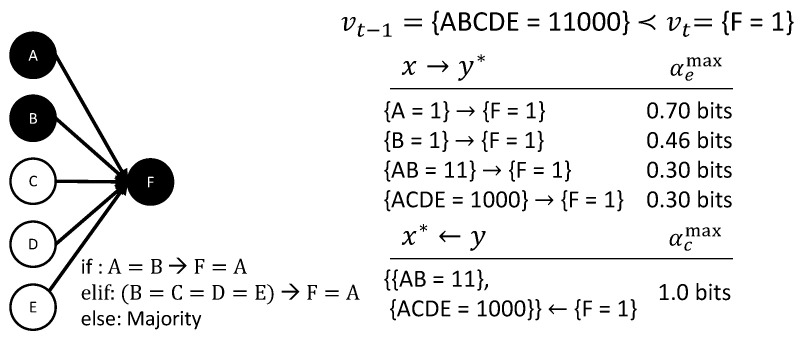
Complicated voting.

As has already been demonstrated in the examples in Figure 7C,D, the proposed causal analysis is not restricted to linear update functions or combinations thereof. Figure 10 depicts an example transition featuring a complicated, non-linear update function. This specific example is taken from [12,21]: If *A* and *B* agree, *F* takes their value; if *B*, *C*, *D*, and *E* agree, *F* takes *A*’s value; otherwise, the majority decides. The transition of interest is $v_{t-1}=\left\{ABCDE=11000\right\}≺v_{t}=\left\{F=1\right\}$.

According to [21], intuition suggests that {A=1} together with {B=1} cause {F=1}. Indeed, {AB=11} is one minimally-sufficient occurrence in the transition that determines {F=1}. The result of the present causal analysis of the transition (Figure 10) is that both {AB=11} and {ACDE=1000} completely determine that {F=1} will occur with $\alpha_{c}\left(x_{t-1},y_{t}\right)=\alpha_{c}^{\max}\left(y_{t}\right)=1.0$. Thus, there is indeterminism between these two causes. In addition, the effects {A=1}→{F=1}, {B=1}→{F=1}, {AB=11}→{F=1}, and {ACDE=1000}→{F=1} all contribute to the causal account.

### 3.6. Non-Binary Variables

**Figure 11:**
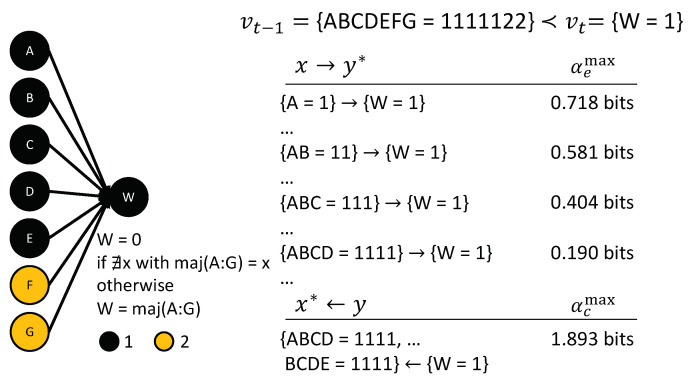
Voting with three possible candidates.

To demonstrate the utility of our proposed framework in the case of non-binary variables, we consider a voting scenario with three possible candidates (“1”, “2”, and “3”), as originally suggested by [48]. Let us assume that there are seven voters, five of which vote in favor of candidate “1”, and the remaining two vote in favor of candidate “2”; therefore, candidate “1” wins (Figure 11). This corresponds to the transition $v_{t-1}=\left\{ABCDEFG=1111122\right\}≺v_{t}=\left\{W=1\right\}$. A simple majority is sufficient for any candidate to win. The winner is indicated by {W=1/2/3}, respectively. Throughout, we assume that no candidate wins in case of a tie for the maximum number of votes, in which case {W=0}.

If there were only two candidates, this example would reduce to a simple linear threshold unit with n=7 inputs and threshold k=4. To recall, according to Theorem 1, one out of all minimally sufficient sets of 4 voters in favor of candidate “1” would be chosen as the actual cause of {W=1}, for such a binary LTU — which one remains undetermined. However, the fact that there are three candidates changes the mechanistic nature of the example, as the number of votes necessary for winning now depends on the particular input state. While four votes are always sufficient to win, three votes suffice if the other two candidates each receive two votes.

As a result, the example transition {ABCDEFG=1111122}≺{W=1} poses a problem case for certain contingency-based accounts of actual causation, including the HP definition [21], which declares all individual voters as separate causes of {W=1}, including {F=2} and {G=2} [48]. This is because there are certain contingencies under which the votes for other candidates matter for {W=1} (e.g., {ABCDEFG=1112233}≺{W=1}). However, in the transition of interest, there are sufficient votes for “1” to ensure {W=1}, regardless of the state of the other variables. Here, {F=2} and {G=2}, by themselves, decrease the probability of {W=1}. Accordingly, the present causal analysis identifies an undetermined set of four out of the five voters in favor of candidate “1” as the actual cause, as in the binary case, but with $\alpha_{c}^{max}=1.893$, while $\alpha_{c/e}=0$ for {F=2} and {G=2}. Figure 11 shows the causal account of the transition of interest. All input sets equivalent to the listed occurrences also have an actual effect on {W=1}. By contrast, in the specific case of a 3-2-2 vote ({ABCDEFG=1112233}≺{W=1}), the present account would identify the entire set of inputs as the actual cause of {W=1}; as, in that case, candidate “1” might not have won if any of the votes had been different.

### 3.7. Noise and Probabilistic Variables

**Figure 12:**
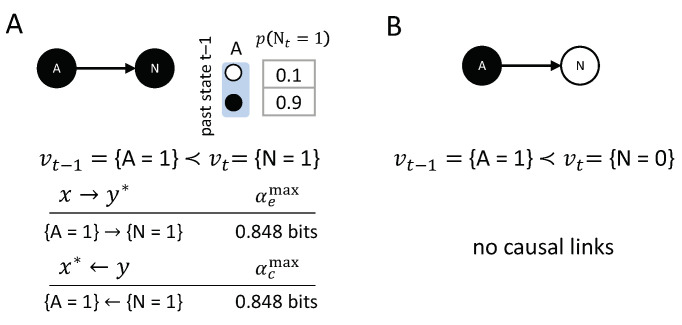
Probabilistic variables. While the transition shown in (**A**) does have a deterministic equivalent, the transition shown in (**B**) would be impossible in the deterministic case.

The examples, so far, have involved deterministic update functions. Probabilistic accounts of causation are closely related to counterfactual accounts [10]. Nevertheless, certain problem cases only arise in probabilistic settings (e.g., that of Figure 12B). The present causal analysis can be applied equally to probabilistic and deterministic causal networks, as long as the system’s transition probabilities satisfy conditional independence (Equation (1)). No separate, probabilistic calculus for actual causation is required.

In the simplest case, where noise is added to a deterministic transition $v_{t-1}≺v_{t}$, the noise will generally decrease the strength of the causal links in the transition. Figure 12 shows the causal account of the transition $v_{t-1}=\left\{A=1\right\}≺v_{t}=\left\{N=1\right\}$, where *N* is the slightly noisy version of a COPY-gate. In this example, both {A=1}→{N=1} and {A=1}←{N=1}. The only difference with the equivalent deterministic case is that the causal strength $\alpha_{e}^{\max}=\alpha_{c}^{\max}=0.848$ is lower than in the deterministic case, where $\alpha_{e}^{\max}=\alpha_{c}^{\max}=1$. Note that, in this probabilistic setting, the actual cause {A=1} by itself is not sufficient to determine {N=1}. Nevertheless, {A=1} makes a positive difference in bringing about {N=1}, and this difference is irreducible, so the causal link is present within the transition.

The transition $v_{t-1}=\left\{A=1\right\}≺v_{t}=\left\{N=0\right\}$ has no counterpart in the deterministic case, where p({N=0}|{A=1})=0 (considering the transition would thus violate the realization principle). The result of the causal analysis is that there are no integrated causal links within this transition. We have that {A=1} decreases the probability of {N=0}, and vice versa, which leads to $\alpha_{c/e}<0$. Consequently, $\alpha_{c/e}^{\max}=0$, as specified by the empty set. One interpretation is that the actual cause of {N=0} must lie outside of the system, such as a missing latent variable. Another interpretation is that the actual cause for {N=0} is genuine ‘physical noise’; for example, within an element or connection. In any case, the proposed account of actual causation is sufficiently general to cover both deterministic, as well as probabilistic, systems.

### 3.8. Simple Classifier

As a final example, we consider a transition with a multi-variate $v_{t}$: The three variables *A*, *B*, and *C* provide input to three different “detectors”, the nodes *D*, *S*, and *L*. *D* is a “dot-detector”: It outputs ‘1’ if exactly one of the 3 inputs is in state ‘1’; *S* is a “segment-detector”: It outputs ‘1’ for input states {ABC=110} and {ABC=011}; and *L* detects lines—that is, {ABC=111}.

**Figure 13:**
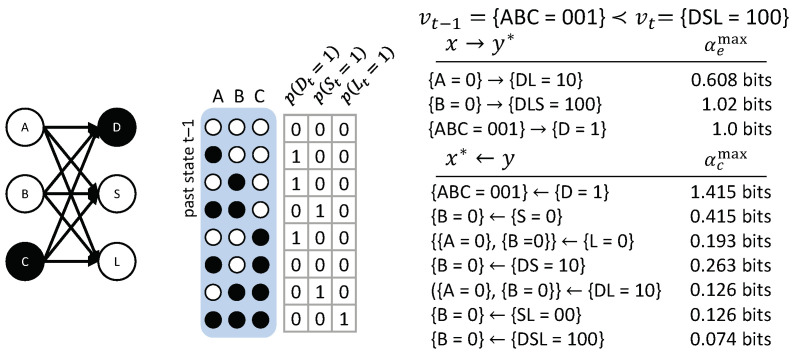
Simple classifier. *D* is a “dot-detector”, *S* is a “segment-detector”, and *L* is a “line-detector” (see text).

Figure 13 shows the causal account of the specific transition $v_{t-1}=\left\{ABC=001\right\}≺v_{t}=\left\{DSL=100\right\}$. In this case, only a few occurrences $x_{t-1}\subseteq v_{t-1}$ have actual effects, but all possible occurrences $y_{t}\subseteq v_{t}$ are irreducible with their own actual cause. The occurrence {C=1} by itself, for example, has no actual effect. This may be initially surprising, since *D* is a dot detector and {C=1} is, supposedly, a dot. However, {C=1} by itself does not raise the probability of {D=1}. The specific configuration of the entire input set is necessary to determine {D=1} (a dot is only a dot if the other inputs are ‘0’). Consequently, {ABC=001}→{D=1} and also {ABC=001}←{D=1}. By contrast, the occurrence {A=0} is sufficient to determine {L=0} and raises the probability of {D=1}; the occurrence {B=0} is sufficient to determine {S=0} and {L=0} and also raises the probability of {D=1}. We, thus, get the following causal links: {A=0}→{DL=10}, {{A=0},{B=0}}←{L=0}, {B=0}→{DSL=100}, and {B=0}←{S=0}.

In addition, all high-order occurrences $y_{t}$ are irreducible, each having their own actual cause above those of their parts. The actual cause identified for these high-order occurrences can be interpreted as the “strongest” shared cause of nodes in the occurrence; for example, {B=0}←{DS=10}. While only the occurrence {ABC=001} is sufficient to determine {DS=10}, this candidate causal link is reducible, because {DS=10} does not constrain the past state of ABC any more than {D=1} by itself. In fact, the occurrence {S=0} does not constrain the past state of AC at all. Thus, {ABC=001} and all other candidate causes of {DS=10} that include these nodes are either reducible (because their causal link can be partitioned with $\alpha_{c}^{\max}=0$) or excluded (because there is a subset of nodes whose causal strength is at least as high). In this example, {B=0} is the only irreducible shared cause of {D=1} and {S=0}, and, thus, is also the actual cause of {DS=10}.

## 4. Discussion

In this article, we presented a principled, comprehensive formalism to assess actual causation within a given dynamical causal network $G_{u}$. For a transition $v_{t-1}≺v_{t}$ in $G_{u}$, the proposed framework provides a complete causal account of all causal links between occurrences at t−1 and *t* of the transition, based on five principles: Realization, composition, information, integration, and exclusion. In what follows, we review specific features and limitations of our approach, discuss how the results relate to intuitive notions about actual causation and causal explanation, and highlight some of the main differences with previous proposals aimed at operationalizing the notion of actual causation. Specifically, our framework considers all counterfactual states, rather than a single contingency, which makes it possible to assess the strength of causal links. Second, it distinguishes between actual causes and actual effects, which are considered separately. Third, it allows for causal composition, in the sense that first- and high-order occurrences can have their own causes and effects within the same transition, as long as they are irreducible. Fourth, it provides a rigorous treatment of causal overdetermination. As demonstrated in the results section, the proposed formalism is generally applicable to a vast range of physical systems, whether deterministic or probabilistic, with binary or multi-valued variables, feedforward or recurrent architectures, as well as narrative examples; as long as they can be represented as a causal network with an explicit temporal order.

### 4.1. Testing All Possible Counterfactuals with Equal Probability

In the simplest case, counterfactual approaches to actual causation are based on the “but-for” test [12]: C=c is a cause of E=e if C=¬c implies E=¬e (“but for *c*, *e* would not have happened”). In multi-variate causal networks, this condition is typically dependent on the remaining variables *W*. What differs among current counterfactual approaches are the permissible *contingencies* (W=w) under which the “but-for” test is applied (e.g., [8,11,19,20,21,30,31]). Moreover, if there is one permissible contingency (counterfactual state) {¬c,w} that implies E=¬e, then *c* is identified as a cause of *e* in an “all-or-nothing” manner. In summary, current approaches test for counterfactual dependence under a fixed contingency W=w, evaluating a particular counterfactual state C=¬c. This holds true, even for recently-proposed extensions of contingency-based accounts of actual causation to probabilistic causal models [49,50] (see, however, [51] for an alternative approach, based on CP-logic).

**Figure 14:**
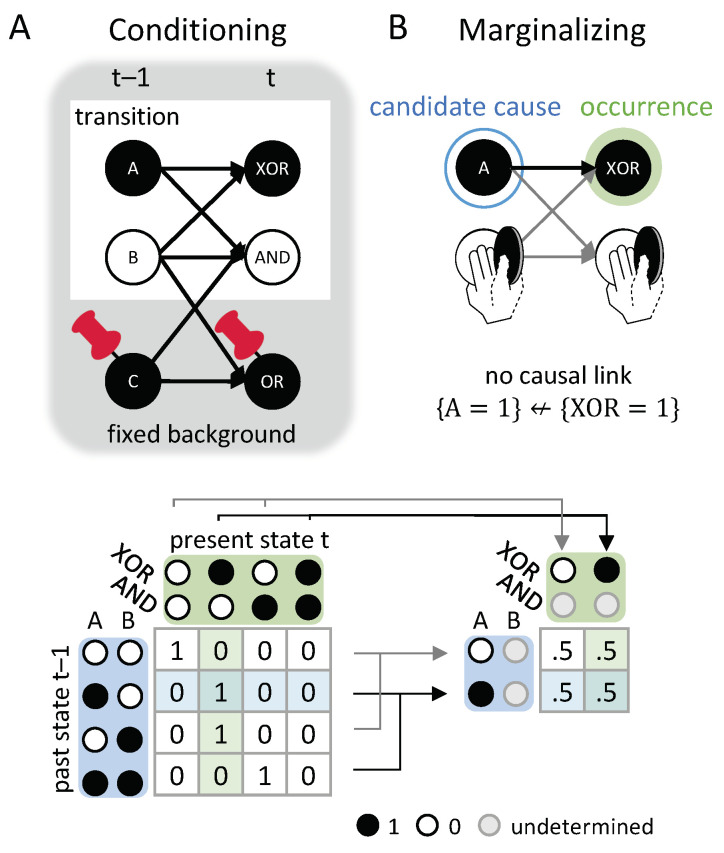
Causal conditioning and marginalizing. (**A**) Variables outside the transition of interest are treated as fixed background conditions (indicated by the red pins). The transition probabilities $p(v_{t}|v_{t-1})$ are conditioned on the state of these background variables. (**B**) When evaluating the strength of a causal link within the transition, the remaining variables in $G_{u}$ outside the causal link are causally marginalized; that is, they are replaced by an average across all their possible states. With *B* marginalized, the state of *A* by itself does not determine and is not determined by the occurrence {XOR=1}.

Our starting point is a realization of a dynamical causal network $G_{u}$, which is a transition $v_{t-1}≺v_{t}$ that is compatible with $G_{u}$’s transition probabilities ($p_{u}\left(v_{t}|v_{t-1}\right)>0$) given the fixed background conditions U=u (Figure 14A). However, we employ *causal marginalization*, instead of fixed W=w and C=¬c, within the transition. This means that we replace these variables with an average over *all* their possible states (see Equation (2)).

Applied to variables outside of the candidate causal link (see Figure 14B), causal marginalization serves to remove the influence of these variables on the causal dependency between the occurrence and its candidate cause (or effect), which is, thus, evaluated based on its own merits. The difference between marginalizing the variables outside the causal link of interest and treating them as fixed contingencies becomes apparent in the case of the XOR (“exclusive OR”) mechanism in Figure 14 (or, equivalently, the bi-conditional (XNOR) in Figure 7C). With the input *B* fixed in a particular state (‘0’ or ‘1’), the state of the XOR will completely depend on the state of *A*. However, the state of *A* alone does not determine the state of the XOR at all if *B* is marginalized. The latter better captures the mechanistic nature of the XOR, which requires a difference in *A* and *B* to switch on (‘1’).

We also marginalize across all possible states of *C*, in order to determine whether *e* counterfactually depends on *c*. Instead of identifying one particular C=¬c for which E=¬e, all of *C*’s states are equally taken into account. The notion that counterfactual dependence is an “all-or-nothing concept” [12] becomes problematic; for example, if non-binary variables are considered, and also in non-deterministic settings. By contrast, our proposed approach, which considers all possible states of *C*, naturally extends to the case of multi-valued variables and probabilistic causal networks. Moreover, it has the additional benefit that we can quantify the strength of the causal link between an occurrence and its actual cause (effect). In the present framework, having positive effect information $\rho_{e}\left(x_{t-1},y_{t}\right)>0$ is necessary, but not sufficient, for $x_{t-1}\to y_{t}$, and the same for positive cause information $\rho_{c}\left(x_{t-1},y_{t}\right)>0$.

Taken together, we argue that causal marginalization—that is, averaging over contingencies and all possible counterfactuals of an occurrence—reveals the mechanisms underlying the transition. By contrast, fixing relevant variables to any one specific state largely ignores them. This is because a mechanism is only fully described by all of its transition probabilities, for all possible input states (Equation (1)). For example, the bi-conditional *E* (in Figure 7C) only differs from the conjunction *D* (in Figure 7B) for the input state AB = 00. Once the underlying mechanisms are specified, based on all possible transition probabilities, causal interactions can be quantified in probabilistic terms [25,32], even within a single transition $v_{t-1}≺v_{t}$ (i.e., in the context of actual causation [33,52]). However, this also means that all transition probabilities have to be known for the proposed causal analysis, even for states that are not typically observed (see also [25,32,34,42]).

Finally, in our analysis, all possible past states are weighted equally in the causal marginalization. Related measures of information flow in causal networks [32], causal influence [34], and causal information [33] consider weights based on a distribution of $p(v_{t-1})$; for example, the stationary distribution, observed probabilities, or a maximum entropy distribution (equivalent to weighting all states equally). Janzing et al. [34], for example, proposed to quantify the “factual” direct causal influence across a set of edges in a causal network by “cutting” those edges, and comparing the joint distribution before and after the cut. Their approach is very similar to our notion of partitioning. However, instead of weighting all states equally in the marginalization, they marginalized each variable according to its probabilities in the joint distribution, which typically depend on the long-term dynamics of the system (and, thus, on other mechanisms within the network than the ones directly affected by the cut), as well as the state in which the system was initialized. While this makes sense for a measure of *expected* causal strength, in the context of actual causation the prior probabilities of occurrences at t−1 are extraneous to the question “what caused what?” All that matters is what actually happened, the transition $v_{t-1}≺v_{t}$, and the underlying mechanisms. How likely $v_{t-1}$ was to occur should not influence the causes and effects within the transition, nor how strong the causal links are between actual occurrences at t−1 and *t*. In other words, the same transition, involving the same mechanisms and background conditions, should always result in the same causal account. Take, for instance, a set of nodes A,B that output to *C*, which is a deterministic OR-gate. If *C* receives no further inputs from other nodes, then whenever {AB=11} and {C=1}, the causal links, their strength, and the causal account of the transition {AB=11}≺{C=1} should be the same as in Figure 7A (“Disjunction”). Which larger system the set of nodes was embedded in, or what the probability was for the transition to happen in the first place, according to the equilibrium, observed, or any other distribution, is not relevant in this context. Let us assume, for example, that {A=1} was much more likely to occur than {B=1}. This bias in prior probability does not change the fact that, mechanistically, {A=1} and {B=1} have the same effect on {C=1}, and are equivalent causes.

### 4.2. Distinguishing Actual Effects and Actual Causes

An implicit assumption, commonly made about (actual) causation, is that the relation between cause and effect is bidirectional: If occurrence C=c had an effect on occurrence E=e, then *c* is assumed to be a cause of *e* [8,11,19,20,21,30,31,49,50]. As demonstrated throughout the Results section, however, this conflation of causes and effects is untenable, once multi-variate transitions $v_{t-1}≺v_{t}$ are considered (see also Section 4.3 below). There, an asymmetry between causes and effects simply arises, due to the fact that the set of variables that is affected by an occurrence $x_{t-1}\subseteq v_{t-1}$ typically differs from the set of variables that affects an occurrence $y_{t}\subseteq v_{t}$. Take the toy classifier example in Figure 13: While {B=0} is the actual cause of {S=0}, the actual effect of {B=0} is {DLS=100}.

Accordingly, we propose that a comprehensive causal understanding of a given transition is provided by its complete causal account C (Definition 4), including both actual effects and actual causes. Actual effects are identified from the perspective of occurrences at t−1, whereas actual causes are identified from the perspective of occurrences at *t*. This means that also the causal principles of composition, integration, and exclusion are applied from these two perspectives. When we evaluate causal links of the form $x_{t-1}\to y_{t}$, any occurrence $x_{t-1}$ may have one actual effect $y_{t}\subseteq v_{t}$ if $x_{t-1}$ is irreducible ($\alpha_{e}^{\max}\left(x_{t-1}\right)>0$) (Definition 2). When we evaluate causal links of the form $x_{t-1}\leftarrow y_{t}$, any occurrence $y_{t}$ may have one actual cause $x_{t}\subseteq v_{t-1}$ if $y_{t}$ is irreducible ($\alpha_{c}^{\max}\left(y_{t}\right)>0$) (Definition 1). As seen in the first example (Figure 6), there may be a high-order causal link in one direction, but the reverse link may be reducible.

As mentioned in the Introduction and exemplified in the Results, our approach has a more general scope, but is still compatible with the traditional view of actual causation, concerned only with actual causes of singleton occurrences. Nevertheless, even in the limited setting of a singleton $v_{t}$, considering both causes and effects may be illuminating. Consider, for example, the transition shown in Figure 9A: By itself, the occurrence {A=1} raises the probability of {D=1} ($\rho_{e}\left(x_{t-1},y_{t}\right)=\alpha_{e}\left(x_{t-1},y_{t}\right)>0$), which is a common determinant of being a cause in probabilistic accounts of (actual) causation [13,14,53,54] (Note though that Pearl initially proposed maximizing the posterior probability p(c∣e) as a means of identifying the best (“most probable”) explanation for an occurrence *e* ([16]; Chapter 5). However, without a notion of irreducibility, as applied in the present framework, explanations based on p(c∣e) tend to include irrelevant variables [29,55]). Even in deterministic systems with multi-variate dependencies, however, the fact that an occurrence *c*, by itself, raises the probability of an occurrence *e*, does not necessarily determine that E=e will actually occur [10]. In the example of Figure 9, {A=1} is neither necessary nor sufficient for {D=1}. Here, this issue is resolved by acknowledging that both {A=1} and {C=1} have an actual effect on {D=1}, whereas {C=1} is identified as the (one) actual cause of {D=1}, in line with intuition [21].

In summary, an actual effect $x_{t-1}\to y_{t}$ does not imply the corresponding actual cause $x_{t-1}\leftarrow y_{t}$, and vice versa. Including both directions in the causal account may, thus, provide a more comprehensive explanation of “what happened” in terms of “what caused what”.

### 4.3. Composition

The proposed framework of actual causation explicitly acknowledges that there may be high-order occurrences which have genuine actual causes or actual effects. While multi-variate dependencies play an important role in complex distributed systems [4,5,56], they are largely ignored in the actual causation literature.

From a strictly informational perspective focused on predicting $y_{t}$ from $x_{t-1}$, one might be tempted to disregard such compositional occurrences and their actual effects, since they do not add predictive power. For instance, the actual effect of {AB=11} in the conjunction example of Figure 7B is informationally redundant, since {D=1} can be inferred (predicted) from {A=1} and {B=1} alone. From a causal perspective, however, such compositional causal links specify mechanistic constraints that would not be captured, otherwise. It is these mechanistic constraints, and not predictive powers, that provide an explanation for “what happened” in the various transitions shown in Figure 7, by revealing “what caused what”. In Figure 7C, for example, the individual nodes *A* and *B* do not fulfill the most basic criterion for having an effect on the XNOR node {E=1}, as $\rho_{e}\left(x_{t-1},y_{t}\right)=0$; whereas the second-order occurrence {AB=11} has the actual effect {E=1}. In the conjunction example (Figure 7B), {A=1} and {B=1} both constrain the AND-gate *D* in the same way, but the occurrence {AB=11} further raises the probability of {D=1} compared to the effect of each individual input. The presence of causal links specified by first-order occurrences does not exclude the second-order occurrence {AB=11} from having an additional effect on {D=1}.

**Figure 15:**
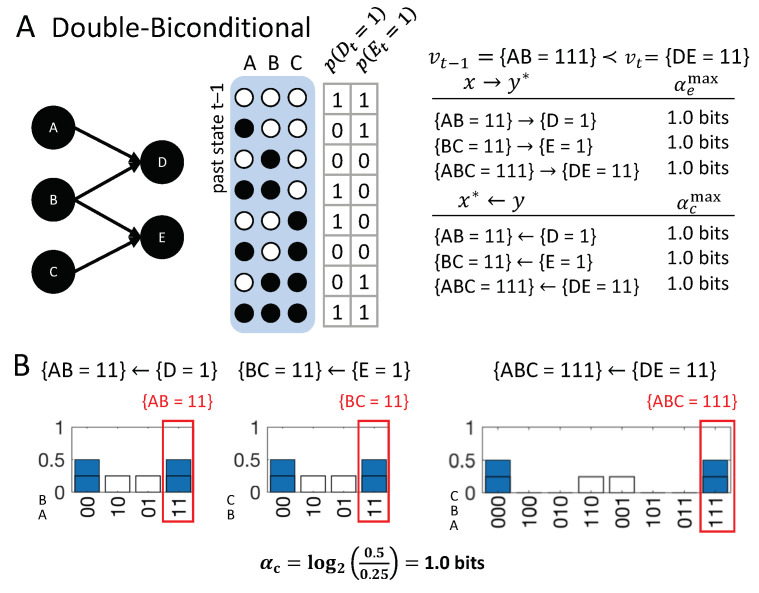
Composition: High-order occurrences. (**A**) Double Bi-conditional: Transition and causal account. (**B**) Cause repertoires corresponding to the two first-order and one second-order occurrences with actual causes (see text).

To illustrate this, with respect to both actual causes and actual effects, we can extend the XNOR example to a “double bi-conditional” and consider the transition $v_{t-1}=\left\{ABC=111\right\}≺v_{t}=\left\{DE=11\right\}$ (see Figure 15). In the figure, both *D* and *E* are XNOR nodes that share one of their inputs (node *B*), and {AB=11}←{D=1} and {BC=11}←{E=1}. As illustrated by the cause-repertoires shown in Figure 15B, and in accordance with *D*’s and *E*’s logic function (mechanism), the actual cause of {D=1} can be described as the fact that *A* and *B* were in the same state, and the actual cause of {E=1} as the fact that *B* and *C* were in the same state. In addition to these first-order occurrences, also the second-order occurrence {DE=11} has an actual cause {ABC=111}, which can be described as the fact that all three nodes *A*, *B*, and *C* were in the same state. Crucially, this fact is not captured by either the actual cause of {D=1}, or by the actual cause of {E=1}, but only by the constraints of the second-order occurrence {DE=11}. On the other hand, the causal link {ABC=111}←{DE=11} cannot capture the fact that {AB=11} was the actual cause of {D=1} and {BC=11} was the actual cause of {E=1}. It is of note, in this example, that the same reasoning applies to the composition of high-order occurrences at t−1 and their actual effects.

In summary, high-order occurrences capture multi-variate mechanistic dependencies between the occurrence variables that are not revealed by the actual causes and effects of their parts. Moreover, a high-order occurrence does not exclude lower-order occurrences over their parts, which specify their own actual causes and effects. In this way, the composition principle makes explicit that high-order and first-order occurrences all contribute to the explanatory power of the causal account.

### 4.4. Integration

**Figure 16:**
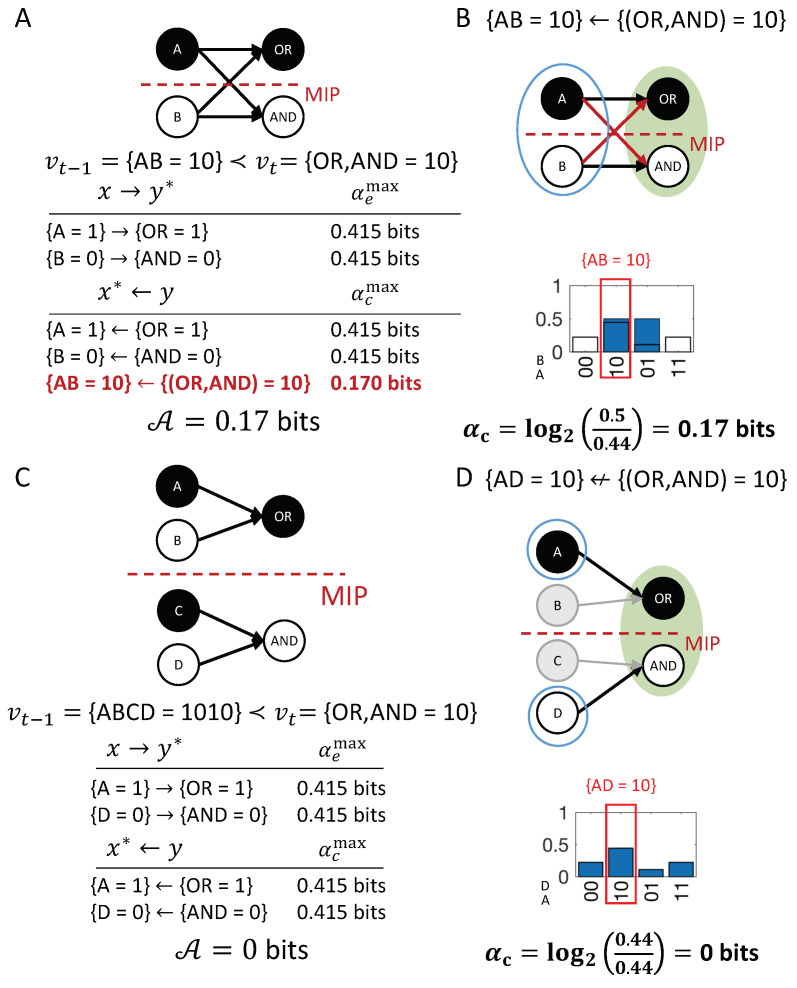
Integration: Irreducible versus reducible occurrences. (**A**) Transition and causal account of Figure 6. (**B**) The second-order occurrence {(OR,AND)=10} with actual cause {AB=10} is irreducible under the MIP. (**C**) Reducible transition with equivalent first-order causal links, but missing the second-order causal link present in (**A**). (**D**) The constraints specified by the second-order occurrence {(OR,AND)=10} here are the same, and thus reducible, to those under the MIP.

As discussed above, high-order occurrences can have actual causes and effects, but only if they are irreducible to their parts. This is illustrated in Figure 16, in which a transition equivalent to our initial example in Figure 6 (Figure 16A) is compared against a similar, but reducible transition (Figure 16C) in a different causal network. The two situations differ mechanistically: The OR and AND gates in Figure 16A receive common inputs from the same two nodes, while the OR and AND in Figure 16C have independent sets of inputs. Nevertheless, the actual causes and effects of all single-variable occurrences are identical in the two cases. In both transitions, {OR=1} is caused by its one input in state ‘1’, and {AND=0} is caused by its one input in state ‘0’. What distinguishes the two causal accounts is the additional causal link, in Figure 16A, between the second-order occurrence {(OR,AND)=10} and its actual cause {AB=10}. Furthermore, {(OR,AND)=10} raises the probability of both {AB=10} (in Figure 16A) and {AD=10} (in Figure 16C), compared to their unconstrained probability π=0.25 and, thus, $\rho_{c}\left(x_{t-1},y_{t}\right)>0$ in both cases. Yet, only {AB=10}←{(OR,AND)=10} in Figure 16A is irreducible to its parts. This is shown by partitioning across the MIP with $\alpha_{c}\left(x_{t-1},y_{t}\right)=0.17$. This second-order occurrence, thus, specifies that the OR and AND gates in Figure 16A receive common inputs—a fact that would, otherwise, remain undetected.

As described in Appendix A, using the measure $A(v_{t-1}≺v_{t})$, we can also quantify the extent to which the entire causal account C of a transition $v_{t-1}≺v_{t}$ is irreducible. The case where $A(v_{t-1}≺v_{t})=0$ indicates that $v_{t-1}≺v_{t}$ can either be decomposed into multiple transitions without causal links between them (e.g., Figure 16C), or includes variables without any causal role in the transition (e.g., Figure 7D).

### 4.5. Exclusion

**Figure 17:**
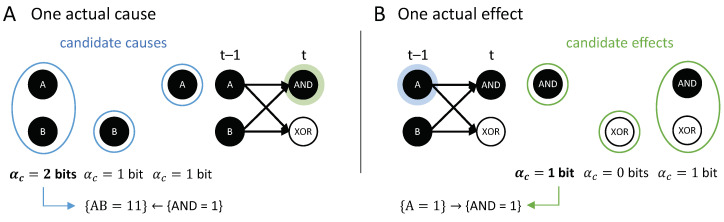
Exclusion: Any occurrence can, at most, have one actual cause or effect. (**A**) Out of the three candidate causes {A=1}, {B=1}, and {AB=11}, the actual cause of {AND=1} is the high-order occurrence {AB=11}, with $\alpha_{c}=\alpha_{c}^{\max}=2.0$ bits. (**B**) Out of the three candidate effects, {AND=1}, {XOR=1}, and {(AND,XOR)=11}, the actual effect of {A=1} is the first-order occurrence {AND=1}, with $\alpha_{e}=\alpha_{e}^{\max}=1.0$ bit; {(AND,XOR)=11} is excluded by the minimality condition (Definition 2).

That an occurrence can affect several variables (high-order effect), and that the cause of an occurrence can involve several variables (high-order cause) is un-controversial [57]. Nevertheless, the possibility of multi-variate causes and effects is rarely addressed in a rigorous manner. Instead of one high-order occurrence, contingency-based approaches to actual causation typically identify multiple first-order occurrences as separate causes in these cases. This is because some approaches only allow for first-order causes by definition (e.g., [11]), while other accounts include a minimality clause that does not consider causal strength and, thus, excludes virtually all high-order occurrences in practice (e.g., [20]; but see [21]). Take the example of a simple conjunction AND=A∧B in the transition {AB=11}≺{AND=1} (see Figure 7B and Figure 17). To our knowledge, all contingency-based approaches regard the first-order occurrences {A=1} and {B=1} as two separate causes of {AND=1}, in this case (but see [58]); while we identify the second-order occurrence {AB=11} (the conjunction) as the one actual cause, with $\alpha_{c}^{\max}$.

Given a particular occurrence, $x_{t-1}$, in the transition $v_{t-1}≺v_{t}$, we explicitly consider the whole power set of $v_{t}$ as candidate effects of $x_{t-1}$, and the whole power set of $v_{t-1}$ as candidate causes of a particular occurrence $y_{t}$ (see Figure 17). However, the possibility of genuine multi-variate actual causes and effects requires a principled treatment of causal over-determination. While most approaches to actual causation generally allow both {A=1} and {B=1} to be actual causes of {AND=1}, this seemingly-innocent violation of the causal exclusion principle becomes prohibitive once {A=1}, {B=1}, and {AB=11} are recognized as candidate causes. In this case, either {AB=11} was the actual cause, or {A=1}, or {B=1}. Allowing for any combination of these occurrences, however, would be illogical. Within our framework, any occurrence can, thus, have, at most, one actual cause (or effect) within a transition—the minimal occurrence with $\alpha^{\max}$ (Figure 17). Finally, cases of true mechanistic over-determination, due to symmetries in the causal network, are resolved by leaving the actual cause (effect) indetermined between all $x^{*}\left(y_{t}\right)$ with $\alpha_{c}^{\max}$ (see Definitions 1 and 2). In this way, the causal account provides a complete picture of the actual mechanistic constraints within a given transition.

### 4.6. Intended Scope and Limitations

The objective of many existing approaches to actual causation is to provide an account of people’s intuitive causal judgments [12,51]. For this reason, the literature on actual causation is largely rooted in examples involving situational narratives, such as “Billy and Suzy throw rocks at a bottle” [7,12], which are then compressed into a causal model to be investigated. Such narratives can serve as intuition pumps, but can also lead to confusion if important aspects of the story are omitted in the causal model applied to the example [9,10,11].

Our objective is to provide a principled, quantitative causal account of “what caused what” within a fully-specified (complete) model of a physical systems of interacting elements. We purposely set aside issues regarding model selection or incomplete causal knowledge, in order to formulate a rigorous theoretical framework applicable to any pre-determined dynamical causal network [12,36]. This puts the explanatory burden on the formal framework of actual causation, rather than on the adequacy of the model. In this setting, causal models should always be interpreted mechanistically and time is explicitly taken into account. Rather than on capturing intuition, an emphasis is put on explanatory power and consistency (see, also, [10]). With a proper formalism in place, future work should address to what extent and under which conditions the identified actual causes and effects generalize across possible levels of description (macro versus micro causes and effects), or under incomplete knowledge (see, also, [38,39]). While the proposed theoretical framework assumes idealized conditions and an exhaustive causal analysis is only feasible in rather small systems, a firm theoretical basis should facilitate the development of consistent empirical approximations for assessing actual causation in practice (see, also, [7,34]).

In addition, the examples examined in this study have been limited to direct causes and effects within transitions $v_{t-1}≺v_{t}$ across a single system update. The explanatory power of the proposed framework was illustrated in several examples, which included paradigmatic problem cases involving overdetermination and prevention. Yet, some prominent examples that raise issues of “pre-emption” or “causation by omission” have no direct equivalent in these basic types of physical causal models. While the approach can, in principle, identify and quantify counterfactual dependencies across k>1 time steps by replacing $p_{u}\left(v_{t}|v_{t-1}\right)$ with $p_{u}\left(v_{t}|v_{t-k}\right)$ in Equation (1), for the purpose of tracing a causal chain back in time [58], the role of intermediary occurrences remains to be investigated. Nevertheless, the present framework is unique in providing a general, quantitative, and principled approach to actual causation that naturally extends beyond simple, binary, and deterministic example cases, to all mechanistic systems that can be represented by a set of transition probabilities (as specified in Equation (1)).

### 4.7. Accountability and Causal Responsibility

This work presents a step towards a quantitative causal understanding of “what is happening” in systems such as natural or artificial neural networks, computers, and other discrete, distributed dynamical systems. Such causal knowledge can be invaluable, for example, to identify the reasons for an erroneous classification by a convolutional neural network [59], or the source of a protocol violation in a computer network [60]. A notion of multi-variate actual causes and effects, in particular, is crucial for addressing questions of accountability, or sources of network failures [12] in distributed systems. A better understanding of the actual causal links that govern system transitions should also improve our ability to effectively control the dynamical evolution of such systems and to identify adverse system states that would lead to unwanted system behaviors.

Finally, a principled approach to actual causation in neural networks may illuminate the causes of an agent’s actions or decisions (biological or artificial) [61,62,63], including the causal origin of voluntary actions [64]. However, addressing the question “who caused what?”, as opposed to “what caused what”, implies modeling an agent with intrinsic causal power and intention [60,65]. Future work will extend the present mechanistic framework for “extrinsic” actual causation with a mechanistic account of “intrinsic” actual causation in autonomous agents [25,66].

## 5. Conclusions

We have presented a principled, comprehensive formalism to assess actual causation within a given dynamical causal network $G_{u}$, which can be interpreted as consecutive time steps of a discrete dynamical system (feed-forward or recurrent). Based on five principles adopted from integrated information theory (IIT) [25,27]—realization, composition, information, integration, and exclusion—the proposed framework provides a quantitative causal account of all causal links between occurrences (including multi-variate dependencies) for a transition $v_{t-1}≺v_{t}$ in $G_{u}$.

The strength of a causal link between an occurrence and its actual cause (or effect) is evaluated in informational terms, comparing interventional probabilities before and after a partition of the causal link, which replaces the state of each partitioned variable with an average across all its possible states (causal marginalization). Additionally, the remaining variables in $G_{u}$ but outside the causal link are causally marginalized. Rather than a single contingency, all counterfactual states are, thus, taken into account in the causal analysis. In this way, our framework naturally extends from deterministic to probabilistic causal networks, and also from binary to multi-valued variables, as exemplified above.

The generality of the proposed framework, moreover, makes it possible to derive analytical results for specific classes of causal networks, as demonstrated here for the case of linear threshold units and disjunctions of conjunctions. In the absence of analytical results, the actual cause (or effect) of an occurrence within $G_{u}$ can be determined based on an exhaustive search. Software to evaluate the causal account of simple binary networks (deterministic and probabilistic) is available within the PyPhi software package [44]. While approximations will have to be developed in order to apply our framework to larger systems and empirical settings, our objective here was to lay the theoretical foundation for a general approach to actual causation that allows moving beyond intuitive toy examples to scientific problems where intuition is lacking, such as understanding actual causation in biological or artificial neural networks.

## Figures and Tables

**Figure A1:**
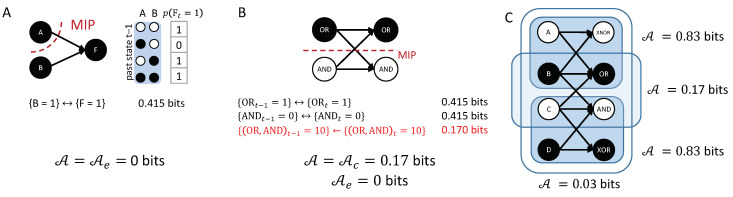
Reducible and irreducible causal accounts. (**A**) “Prevention” example (see Figure 7D, main text). We have that A=0 bits as {A=1} does not contribute to any causal links. (**B**) Irreducible transition (see Figure 6, main text). A partition of the transition along the MIP destroys the 2nd-order causal link, leading to A=0.17 bits. (**C**) In larger systems, A can be used to identify (sub-)transitions with highly entangled causes and effect. While the causes and effects in the full transition are only weakly entangled, with A=0.03 bits, the top and bottom (sub-)transitions are irreducible, with A=0.83 bits.

## Appendix A. Irreducibility of the Causal Account

Similar to the notion of system-level integration in integrated information theory (IIT) [25,26], the principle of integration can also be applied to the causal account as a whole, not only to individual causal links. The causal account of a particular transition $v_{t-1}≺v_{t}$ of the dynamical causal network $G_{u}$ is defined as the set of all causal links within the transition (Definition 4, main text).

In the following, we define the quantity $A(v_{t-1}≺v_{t})$, which measures to what extent the transition $v_{t-1}≺v_{t}$ is irreducible to its parts. Moreover, we introduce $A_{e}\left(v_{t-1}≺v_{t}\right)$, which measures the irreducibility of $v_{t-1}$ and its set of “effect” causal links $\left\{x_{t-1}\to y_{t}\right\}\in C\left(v_{t-1}≺v_{t}\right)$, and $A_{c}\left(v_{t-1}≺v_{t}\right)$, which measures the irreducibility of $v_{t}$ and its set of “cause” causal links $\left\{x_{t-1}\leftarrow y_{t}\right\}\in C\left(v_{t-1}≺v_{t}\right)$. In this way, we can:

- Identify irrelevant variables within a causal account that do not contribute to any causal link (Figure A1A);
- evaluate how entangled the sets of causes and effects are within a transition $v_{t-1}≺v_{t}$ (Figure A1B); and
- compare A values between (sub-)transitions, in order to identify clusters of variables whose causes and effects are highly entangled, or only minimally connected (Figure A1C).

We can assess the irreducibility of $v_{t-1}$ and its set of “effect” causal links $\left\{x_{t-1}\to y_{t}\right\}\in C\left(v_{t-1}≺v_{t}\right)$ in parallel to $\alpha_{e}\left(x_{t-1},y_{t}\right)$, by testing all possible partitions $\Psi (v_{t-1},V_{t})$ (Equation (7)). This means that the transition $v_{t-1}≺v_{t}$ is partitioned into independent parts, in the same manner that an occurrence $x_{t-1}$ is partitioned when assessing $\alpha_{e}\left(x_{t-1},y_{t}\right)$. We, then, define the irreducibility of $v_{t-1}$ as the difference in the total strength of actual effects (causal links of the form $x_{t-1}\to y_{t}$) in the complete causal account C, compared to the causal account under the MIP; which, again, denotes the partition in $\Psi (v_{t-1},V_{t})$ that makes the least difference to C:

(A1) $$A_{e}\left(v_{t-1}≺v_{t}\right)=\sum_{x\to y\in C}\left(\alpha_{e}^{\max}\left(x\right)\right)-\sum_{x\to y\in C_{\mathrm{MIP}}}\left(\alpha_{e}^{\max}{\left(x\right)}_{\mathrm{MIP}}\right).$$

In the same way, the irreducibility of $v_{t}$ and its set of causal links $\left\{x_{t-1}\leftarrow y_{t}\right\}\in C\left(v_{t-1}≺v_{t}\right)$ is defined as the difference in the total strength of actual causes (causal links of the form $x_{t-1}\leftarrow y_{t}$) in the causal account C, compared to the causal account under the MIP:

(A2) $$A_{c}\left(v_{t-1}≺v_{t}\right)=\sum_{x\leftarrow y\in C}\left(\alpha_{c}^{\max}\left(y\right)\right)-\sum_{x\leftarrow y\in C_{\mathrm{MIP}}}\left(\alpha_{c}^{\max}{\left(y\right)}_{\mathrm{MIP}}\right),$$

where the MIP is, again, the partition that makes the least difference out of all possible partitions $\Psi (V_{t-1},v_{t})$ (Equation (9)). This means that the transition $v_{t-1}≺v_{t}$ is partitioned into independent parts in the same manner that an occurrence $y_{t}$ is partitioned when assessing $\alpha_{c}\left(x_{t-1},y_{t}\right)$. The irreducibility of a single-variable $v_{t-1}$ or $v_{t}$ reduces to $\alpha_{e}^{\max}$ of its one actual effect $y_{t}$, or $\alpha_{c}^{\max}$ of its one actual cause $x_{t-1}$, respectively.

By considering the union of possible partitions, $\Psi \left(v_{t-1}≺v_{t}\right)=\Psi \left(v_{t-1},V_{t}\right)\cup \Psi \left(V_{t-1},v_{t}\right)$, we can moreover assess the overall irreducibility of the transition $v_{t-1}≺v_{t}$. A transition $v_{t-1}≺v_{t}$ is reducible if there is a partition $\psi \in \Psi (v_{t-1}≺v_{t})$, such that the total strength of causal links in $C(v_{t-1}≺v_{t})$ is un-affected by the partition. Based on this notion, we define the irreducibility of a transition $v_{t-1}≺v_{t}$ as:

(A3) $$A\left(v_{t-1}≺v_{t}\right)=\sum \alpha^{\max}\left(C\right)-\sum \alpha^{\max}\left(C_{\mathrm{MIP}}\right),$$

where

$$\sum \alpha^{\max}\left(C\right)=\sum_{x\to y\in C}\left(\alpha_{e}^{\max}\left(x\right)\right)+\sum_{x\leftarrow y\in C}\left(\alpha_{c}^{\max}\left(y\right)\right)$$

is a summation over the strength of all causal links in the causal account $C(v_{t-1}≺v_{t})$, and the same for the partitioned causal account $C_{\mathrm{MIP}}$.

Figure A1A shows the “Prevention” example of Figure 7D, main text, where {A=1} has no effect and is not a cause in this transition. Replacing {A=1} with an average over all its possible states does not make a difference to the causal account and, thus, $A(v_{t-1}≺v_{t})=0$ in this case. Figure A1B shows the causal account $C_{\mathrm{MIP}}$ of the transition $v_{t-1}≺v_{t}$ with $v_{t-1}=v_{t}=\left\{\mathrm{OR},\mathrm{AND}=10\right\}$ under its MIP into m=2 parts with $A(v_{t-1}≺v_{t})=0.17$. This is the causal strength that would be lost if we treated $v_{t-1}≺v_{t}$ as two separate transitions, $\left\{{\mathrm{OR}}_{t-1}=1\right\}≺\left\{{\mathrm{OR}}_{t}=1\right\}$ and $\left\{{\mathrm{AND}}_{t-1}=0\right\}≺\left\{{\mathrm{AND}}_{t}=0\right\}$, instead of a single one within $G_{u}$.

The irreducibility $A(v_{t-1}≺v_{t})$ provides a measure of how causally “entangled” the variables *V* are during the transition $v_{t-1}≺v_{t}$. In a larger system, we can measure and compare the A values of multiple (sub-)transitions. In Figure A1C, for example, the causes and effects of the full transition are only weakly entangled (A=0.03 bits), while the transitions involving the four upper or lower variables, respectively, are much more irreducible (A=0.83 bits). In this way, $A(v_{t-1}≺v_{t})$ may be a useful quantity when evaluating more parsimonious causal explanations against the complete causal account of the full transition.

## Appendix B. Supplementary Proof 1

The first theorem describes the actual causes and effects for an observation of a linear threshold unit (LTU) $V_{t}=\left\{Y_{t}\right\}$ with *n* inputs and threshold *k*, and its inputs $V_{t-1}$. First, a series of Lemmas are demonstrated, based on transition probabilities $q_{c,j}$ from an effect repertoire: If $X_{t-1}=x_{t-1}\subseteq V_{t-1}=v_{t-1}$ is an occurrence with size $|X_{t-1}|=c$ and *j* of the *c* elements in $X_{t-1}$ are in the ‘ON’ state $\left(\sum_{x\in x_{t-1}}x=j\right)$, then, by Equation (3)

qc,j=π(Yt=1|Xt−1=xt−1)=∑i=k−jn−c12n−cn−ciifj≤kandj>k−(n−c)1ifj≥k0ifj<k−(n−c).

First, we demonstrate that the probabilities $q_{c,j}$ are non-decreasing as the number of ‘ON’ inputs *j* increases, for a fixed size of occurrence *c*, and that there is a specific range of values of *j* and *c*, such that the probabilities are strictly increasing.

**Lemma** **A1.**
*$q_{c,j}\geq q_{c,j-1}$ with $q_{c,j}=q_{c,j-1}$ iff j>k or j<k−(n−c).*

**Proof.** If j>k, then

$$q_{c,j}=q_{c,j-1}=1.$$

If j<k−(n−c), then

$$q_{c,j}=q_{c,j-1}=0.$$

If k−(n−c)≤j≤k, then

qc,j=12n−c∑i=k−jn−cn−ci=12n−c∑i=k−(j−1)n−cn−ci−12n−c=qc,j−1+12n−c>qc,j−1.

 □

Next, we demonstrate two results relating the transition probabilities between occurrences of different sizes:
**Lemma** **A2.** $q_{c,j}=\frac{1}{2}\left(q_{c+1,j}+q_{c+1,j+1}\right)$ for 1≤c<n and 0≤j≤c.

**Proof.** If j≥k, then

$$q_{c,j}=q_{c+1,j}=q_{c+1,j+1}=1,$$

so

$$q_{c,j}=\frac{1}{2}\left(q_{c+1,j}+q_{c+1,j+1}\right)=1.$$

If j=k−1, then

$$q_{c+1,j+1}=1,$$

and

qc,j=qc,k−1=12n−c∑i=1n−cn−ci=12n−c1+∑i=1n−(c+1)n−(c+1)i−1+n−(c+1)i=1212n−(c+1)∑i=1n−(c+1)n−(c+1)i+12n−(c+1)∑i=0n−(c+1)n−(c+1)i=12(qc+1,j+1)=12(qc+1,j+qc+1,j+1).

If j<k−(n−c), then

$$q_{c,j}=q_{c+1,j}=q_{c+1,j+1}=0,$$

so

$$q_{c,j}=\frac{1}{2}\left(q_{c+1,j}+q_{c+1,j+1}\right)=0.$$

If j=k−(n−c) then

$$q_{c+1,j}=0,$$

and

qc,j=12n−c∑i=n−cn−cn−ci=12n−c∑i=n−(c+1)n−(c+1)n−(c+1)i=12(qc+1,j+1+qc+1,j).

Finally, if k−(n−c)+1<j<k−1, then

qc,j=12n−c∑i=k−jn−cn−ci=12n−c1+∑i=k−jn−(c+1)n−ci=12n−c1+∑i=k−jn−(c+1)n−(c+1)i+∑i=k−jn−(c+1)n−(c+1)i−1=12n−c∑i=k−jn−(c+1)n−(c+1)i+1+∑i=k−jn−(c+1)n−(c+1)i−1=12n−c∑i=k−jn−(c+1)n−(c+1)i+∑i=k−(j+1)n−(c+1)n−(c+1)i=12qc+1,j+qc+1,j+1.

 □

**Lemma** **A3.**
*If c<k, then $q_{c,c}<q_{c+1,c+1}$.*

**Proof.** 

$$\begin{array}{ccc} q_{c,c} & = & \frac{1}{2}\left(q_{c+1,c}+q_{c+1,c+1}\right)\;\;(\mathrm{Lemma}\;1.2)\\ & < & \frac{1}{2}\left(q_{c+1,c+1}+q_{c+1,c+1}\right)\;\;(\mathrm{Lemma}\;1.1)\\ & = & q_{c+1,c+1}. \end{array}$$

 □

Finally, we consider a quantity Q(c), the sum of *q* over all possible states for an occurrence of size *c*. The value Q(c) acts as a normalization term when calculating the cause repertoire of occurrence $\left\{Y_{t}=1\right\}$. Here, we demonstrate a relationship between these normalization terms across occurrences of different sizes:

**Lemma** **A4.**
*Let Q(c)=∑j=0ccjqc,j. Then, $Q\left(c\right)=\frac{1}{2}Q\left(c+1\right)$.*

**Proof.** 

Q(c)=∑j=0ccjqc,j=12∑j=0ccjqc+1,j+qc+1,j+1=12∑j=1ccj−1qc+1,j+∑j=0ccjqc+1,j=12qc+1,c+1+qc+1,0+∑j=1cc+1jqc+1,j=12∑j=0c+1c+1jqc+1,j.=12Q(c+1)

 □

Using the above lemmas, we are now in a position to prove the actual causes and actual effects in the causal account of a single LTU in the ‘ON’ state. The causal account for a LTU in the ‘OFF’ state follows, by symmetry.

**Theorem** **A1.**
*Consider a dynamical causal network $G_{u}$, such that $V_{t}=\left\{Y_{t}\right\}$ is a linear threshold unit with n inputs and threshold k≤n, and $V_{t-1}$ is the set of n inputs to $Y_{t}$. For a transition $v_{t-1}≺v_{t-1}$, with $y_{t}=1$ and $\sum v_{t-1}\geq k$, the following hold:*

1. *The actual cause of $\left\{Y_{t}=1\right\}$ is an occurrence $\left\{X_{t-1}=x_{t-1}\right\}$ with $|x_{t-1}|=k$ and $\min(x_{t-1})=1$. Furthermore, the causal strength of the link is*
  $$\alpha_{c}^{\max}\left(y_{t}\right)=k-\log_{2}\left(\sum_{j=0}^{k}q_{k,j}\right)>0;\;\mathrm{and}$$
2. *If $\min(x_{t-1})=1$ and $|x_{t-1}|\leq k$ then the actual effect of $\left\{X_{t-1}=x_{t-1}\right\}$ is $\left\{Y_{t}=1\right\}$ with causal strength*
  $$\alpha_{e}\left(x_{t-1},y_{t}\right)=\log_{2}\left(\frac{q_{c,c}}{q_{c-1,c-1}}\right)>0,$$
  *otherwise $\left\{X_{t-1}=x_{t-1}\right\}$ is reducible ($\alpha_{e}^{\max}\left(x_{t-1}\right)=0$).*

**Proof.** **Part 1:** Consider an occurrence $\left\{X_{t-1}=x_{t-1}\right\}$, such that $|x_{t-1}|=c\leq n$ and $\sum_{x\in x_{t-1}}x=j$. Then, the probability of $x_{t-1}$ in the cause-repertoire of $y_{t}$ is

$$\pi \left(x_{t-1}|y_{t}\right)=\frac{q_{c,j}}{Q(c)}.$$

As $Y_{t}$ is a first-order occurrence, there is only one possible partition, and the causal strength of a potential link is, thus,

$$\alpha_{c}\left(x_{t-1},y_{t}\right)=\rho_{c}\left(x_{t-1},y_{t}\right)=\log_{2}\left(\frac{\pi (x_{t-1}|y_{t})}{\pi (x_{t-1})}\right)=\log_{2}\left(\frac{2^{c}q_{c,j}}{Q(c)}\right).$$

For a fixed value of *c*, the maximum value of causal strength occurs at j=c (since adding ‘ON’ elements can only increase q(c, j), by Lemma A1),

$$\max_{|x_{t-1}|=c}\alpha_{c}\left(x_{t-1},y_{t}\right)=\max_{j}\log_{2}\left(\frac{2^{c}q_{c,j}}{Q(c)}\right)=\log_{2}\left(\frac{2^{c}q_{c,c}}{Q(c)}\right).$$

Applying Lemmas A3 and A4, we see that, across different values of *c*, this maximum is increasing for 0<c<k,

$$\begin{array}{ccc} \max_{|x_{t-1}|=c+1}\alpha_{c}\left(x_{t-1},y_{t}\right)-\max_{|x_{t-1}|=c}\alpha_{c}\left(x_{t-1},y_{t}\right) & = & \log_{2}\left(\frac{2^{c+1}q_{c+1,c+1}}{Q(c+1)}\right)-\log_{2}\left(\frac{2^{c}q_{c,c}}{Q(c)}\right)\\ & = & \log_{2}\left(\frac{2^{c+1}q_{c+1,c+1}Q\left(c\right)}{2^{c}q_{c,c}Q\left(c+1\right)}\right)\\ & = & \log_{2}\left(\frac{q_{c+1,c+1}}{q_{c,c}}\right)\\ & > & 0, \end{array}$$

and that, for k≤c, the causal strength is constant,

$$\begin{array}{ccc} \max_{|x_{t-1}|=c+1}\alpha_{c}\left(x_{t-1},y_{t}\right)-\max_{|x_{t-1}|=c}\alpha_{c}\left(x_{t-1},y_{t}\right) & = & \log_{2}\left(\frac{2^{c+1}q_{c+1,c+1}}{Q(c+1)}\right)-\log_{2}\left(\frac{2^{c}q_{c,c}}{Q(c)}\right)\\ & = & \log_{2}\left(\frac{q_{c+1,c+1}}{q_{c,c}}\right)\\ & = & \log_{2}\left(\frac{1}{1}\right)=0. \end{array}$$

By setting c=j≥k, we find that the maximum causal strength is

$$\alpha_{c}^{\max}\left(y_{t}\right)=\log_{2}\left(\frac{2^{c}q_{c,c}}{Q(c)}\right)=\log_{2}\left(\frac{2^{k}}{Q(k)}\right)=k-\log_{2}\left(\sum_{j=0}^{k}q_{k,j}\right)>0.$$

Any occurrence $x_{t-1}$ with j≥k has maximal causal strength and satisfies condition (1) for being an actual cause,

$$\alpha_{c}\left(x_{t-1},y_{t}\right)=\log_{2}\left(\frac{2^{c}q_{c,j}}{Q(c)}\right)=\log_{2}\left(\frac{2^{k}}{Q(k)}\right)=\alpha_{c}^{\max}\left(y_{t}\right).$$

If c≥k, then there exists a subset $x_{t-1}^{\prime }\subset x_{t-1}$ with $j^{\prime }\geq k$ and $c^{\prime }<c$ such that $x_{t-1}^{\prime }$ also satisfies condition (1) and, thus, $x_{t-1}$ does not satisfy condition (2). However, if j=c=k, then any subset $x_{t-1}^{\prime }$ of $x_{t-1}$ has $j^{\prime }<k$, and so

$$\alpha_{c}\left(x_{t-1}^{\prime },y_{t}\right)=\log_{2}\left(\frac{2^{c^{\prime }}q_{c^{\prime },j^{\prime }}}{Q(c^{\prime })}\right)<\log_{2}\left(\frac{2^{c}}{Q(c)}\right)=\alpha \left(x_{t-1},y_{t}\right).$$

Thus, $x_{t-1}$ satisfies condition (2). Therefore, we have that the actual cause of $y_{t}$ is an occurrence $x_{t-1}$, such that $|x_{t-1}|=k$ and $\min x_{t-1}=1$,

$$x^{*}\left(y_{t}\right)=\{x_{t-1}\subset v_{t-1}|\min x_{t-1}=1,\;\mathrm{and}\;|x_{t-1}\left|=k\right\}.$$

**Part 2:** Again, consider occurrences $X_{t-1}=x_{t-1}$ with $|x_{t-1}|=c$ and $\sum_{x\in x_{t-1}}x=j$. The probability of $y_{t}$ in the effect repertoire of such an occurrence is

π(yt|xt−1)=qc,j=∑i=k−jn−c12n−cn−ciifj≤kandj>k−(n−c)1ifj≥k0ifj<k−(n−c).

As there is only one element in $v_{t}$, the only question is whether or not $x_{t-1}$ is reducible. If it is reducible, it has no actual effect. Otherwise, its actual effect must be $y_{t}$. First, if j<c, then $\exists \;x=0\in x_{t-1}$ and we can define a partition $\psi =\left\{\left\{\left(x_{t-1}-x\right),y_{t}\right\},\left\{x,⌀\right\}\right\}$, such that

$$\pi {\left(y_{t}|x_{t-1}\right)}_{\psi }=\pi \left(y_{t}|\left(x_{t-1}-x\right)\right)\times \pi \left(⌀|x\right)=\pi \left(y_{t}|\left(x_{t-1}-x\right)\right)=q_{c-1,j}$$

and

$$\alpha_{e}\left(x_{t-1},y_{t}\right)\leq \log_{2}\left(\frac{\pi (y_{t}|x_{t-1})}{\pi {\left(y_{t}|x_{t-1}\right)}_{\psi }}\right)=\log_{2}\left(\frac{q_{c,j}}{q_{c-1,j}}\right)\leq 0\;\left(\mathrm{Lemma}1.1/1.2\right),$$

so $x_{t-1}$ is reducible. Next, we consider the case where j=c but c>k. In this case, we define a partition $\psi =\left\{\left\{\left(x_{t-1}-x\right),y_{t}\right\},\left\{x,⌀\right\}\right\}$ (where $x\in x_{t-1}$ is any element), such that

$$\pi {\left(y_{t}|x_{t-1}\right)}_{\psi }=\pi \left(y_{t}|\left(x_{t-1}-x\right)\right)\times \pi \left(⌀|x\right)=\pi \left(y_{t}|\left(x_{t-1}-x\right)\right)=q_{c-1,c-1},$$

and, since c>k,

$$\alpha_{e}\left(x_{t-1},y_{t}\right)\leq \log_{2}\left(\frac{\pi (y_{t}|x_{t-1})}{\pi {\left(y_{t}|x_{t-1}\right)}_{\psi }}\right)=\log_{2}\left(\frac{q_{c,c}}{q_{c-1,c-1}}\right)=\log_{2}\left(\frac{1}{1}\right)=0,$$

and so $x_{t-1}$ is, again, reducible. Finally, we show that, for j=c and c≤k, that $x_{t-1}$ is irreducible with actual effect $\left\{Y_{t}=1\right\}$. All possible partitions of the pair of occurrences can be formulated as $\psi =\left\{\left\{\left(x_{t-1}-x\right),y_{t}\right\},\left\{x,⌀\right\}\right\}$ (where $x\subseteq x_{t-1}$ with |x|=d>0), such that

$$\pi {\left(y_{t}|x_{t-1}\right)}_{\psi }=\pi \left(y_{t}|\left(x_{t-1}-x\right)\right)\times \pi \left(⌀|x\right)=\pi \left(y_{t}|\left(x_{t-1}-x\right)\right)=q_{c-d,c-d},$$

and

$$\alpha_{e}\left(x_{t-1},y_{t}\right)=\min_{\psi }\log_{2}\left(\frac{\pi (y_{t}|x_{t-1})}{\pi {\left(y_{t}|x_{t-1}\right)}_{\psi }}\right)=\min_{d}\log_{2}\left(\frac{q_{c,c}}{q_{c-d,c-d}}\right).$$

The minimum information partition occurs when d=1 (by Lemma A3) and, thus, $\left\{X_{t-1}=x_{t-1}\right\}$ is irreducible with actual effect $\left\{Y_{t}=1\right\}$ and causal strength

$$\alpha_{e}\left(x_{t-1},y_{t}\right)=\log_{2}\left(\frac{q_{c,c}}{q_{c-1,c-1}}\right).$$

 □

## Appendix C. Supplementary Proof 2

The second theorem describes the actual causes and effects for an observation of a disjunction of conjunctions (DOC) $V_{t}=\left\{Y_{t}\right\}$, which is a disjunction of *k* conjunctions, each over $n_{j}$ elements, and its inputs $V_{t-1}={\left\{{\left\{V_{i,j,t-1}\right\}}_{i=1}^{n_{j}}\right\}}_{j=1}^{k}$. The total number of inputs to the DOC element is $n=\sum_{j=1}^{k}n_{j}$. We consider occurrences $x_{t-1}$ that contain $c_{j}\leq n_{j}$ elements from each of the *k* conjunctions, and the total number of elements is $|x_{t-1}|=c=\sum_{j=1}^{k}c_{j}$. To simplify notation, we further define ${\bar{x}}_{j,t-1}={\left\{v_{i,j,t-1}\right\}}_{i=1}^{n_{j}}$, an occurrence with $c_{j}=n_{j}$ and $c_{j^{\prime }}=0$ if $j^{\prime }\neq j$. In other words, ${\bar{x}}_{j,t-1}$ is the set of elements that make up the $j^{th}$ conjunction. First, a series of lemmas are demonstrated, based on the transition probabilities q(s) from an effect repertoire (Equation (3)):

$$q\left(s\right)=\pi \left(Y_{t}=1|x_{t-1}=s\right).$$

To isolate the specific conjunctions, we define $s_{j}\subset x_{t-1}$ to be the state of $X_{t-1}$ within the $j^{th}$ conjunction, and ${\bar{s}}_{j}=\cup_{i=1}^{j}s_{i}\subseteq x_{t-1}$ be the state $X_{t-1}$ within the first *j* conjunctions. For a DOC with *k* conjunctions, we consider occurrences with $c_{j}$ elements from each conjunction, $X_{t-1}={\left\{{\left\{x_{i,j,t-1}\right\}}_{i=1}^{c_{j}}\right\}}_{j=1}^{k}$. In the specific case of a disjunction of two conjunctions,

$$q\left(s_{1},s_{2}\right)=\left\{\begin{array}{cc} 0\; & \mathrm{if}\;\min\left(s_{1}\right)=\min\left(s_{2}\right)=0\\ \frac{1}{2^{n_{1}-c_{1}}}\; & \mathrm{if}\;\min\left(s_{1}\right)=1,\;\min\left(s_{2}\right)=0\\ \frac{1}{2^{n_{2}-c_{2}}}\; & \mathrm{if}\;\min\left(s_{1}\right)=0,\;\min\left(s_{2}\right)=1\\ \frac{2^{n_{1}-c_{1}}+2^{n_{2}-c_{2}}-1}{2^{n_{1}+n_{2}-c_{1}-c_{2}}} & \mathrm{if}\;\min\left(s_{1}\right)=\min\left(s_{2}\right)=1, \end{array}\right.$$

and, in the case of k>2 conjunctions, we define the probability recursively

$$q\left({\bar{s}}_{k-1},s_{k}\right)=\left\{\begin{array}{cc} q({\bar{s}}_{k-1})\; & \mathrm{if}\;\min(s_{k})=0\\ q\left({\bar{s}}_{k-1}\right)+\frac{\left(1-q\left({\bar{s}}_{k-1}\right)\right)}{2^{n_{k}-c_{k}}}\; & \mathrm{if}\;\min(s_{k})=1 \end{array}\right..$$

The first two lemmas demonstrate the effect of adding an additional element to an occurrence. Adding an ‘ON’ input to an occurrence $x_{t-1}$ can never decrease the probability of $\left\{Y_{t}=1\right\}$, while adding an ‘OFF’ input to an occurrence $x_{t-1}$ can never increase the probability of $\left\{Y_{t}=1\right\}$.

**Lemma** **A5.**
*If $\left\{x_{t-1}=s\right\}=\left\{x_{t-1}^{\prime }=s^{\prime },x_{i,j,t-1}=1\right\}$, then $q\left(s^{\prime }\right)\leq q\left(s\right)$.*

**Proof.** The proof is given by induction. We, first, consider the case where k=2. Assume (without loss of generality) that the additional element $x_{i,j,t-1}$ is from the first conjunction ($c_{1}=c_{1}^{\prime }+1$, $c_{2}=c_{2}^{\prime }$). If $\min(s_{1}^{\prime })=0$, then $q\left(s^{\prime }\right)=q\left(s\right)$. If $\min(s_{2}^{\prime })=0$ and $\min(s_{1}^{\prime })=1$, then

$$\frac{q(s^{\prime })}{q(s)}=\frac{2^{n_{1}-\left(c_{1}^{\prime }+1\right)}}{2^{n_{1}-c_{1}^{\prime }}}=\frac{1}{2}<1,$$

so $q\left(s^{\prime }\right)<q\left(s\right)$. Finally, if $\min\left(s_{1}^{\prime }\right)=\min\left(s_{2}^{\prime }\right)=1$, then

$$\frac{q(s^{\prime })}{q(s)}=\frac{2^{n_{1}+n_{2}-\left(c_{1}^{\prime }+1\right)-c_{2}^{\prime }}\left(2^{n_{1}-c_{1}^{\prime }}+2^{n_{2}-c_{2}^{\prime }}-1\right)}{2^{n_{1}+n_{2}-c_{1}^{\prime }-c_{2}^{\prime }}\left(2^{n_{1}-\left(c_{1}^{\prime }+1\right)}+2^{n_{2}-c_{2}^{\prime }}-1\right)}=\frac{2^{n_{1}-c_{1}^{\prime }}+2^{n_{2}-c_{2}^{\prime }}-1}{2^{n_{1}-c_{1}^{\prime }}+2\left(2^{n_{2}-c_{2}^{\prime }}-1\right)}<1.$$

Therefore, when k=2, we have that $q\left(s^{\prime }\right)\leq q\left(s\right)$. Next, we assume the result holds for k−1, $q\left({\bar{s}}_{k-1}^{\prime }\right)\leq q\left({\bar{s}}_{k-1}\right)$ and demonstrate the result for general *k*. Again, assume the additional element is from the first conjunction ($c_{1}=c_{1}^{\prime }+1,c_{j}=c_{j}^{\prime }$ for j>1). If $\min(s_{k})=0$, then

$$\frac{q({\bar{s}}_{k}^{\prime })}{q({\bar{s}}_{k})}=\frac{q({\bar{s}}_{k-1}^{\prime })}{q({\bar{s}}_{k-1})}\leq 1;$$

and, if $\min(s_{k})=1$, then

$$\begin{array}{ccc} \frac{q({\bar{s}}_{k}^{\prime })}{q({\bar{s}}_{k})} & = & \frac{q\left({\bar{s}}_{k-1}^{\prime }\right)+\left(1-q\left({\bar{s}}_{k-1}^{\prime }\right)\right)/2^{n_{k}-c_{k}}}{q\left({\bar{s}}_{k-1}\right)+\left(1-q\left({\bar{s}}_{k-1}\right)\right)/2^{n_{k}-c_{k}}}\\ & = & \frac{\left(2^{n_{k}-c_{k}}-1\right)q\left({\bar{s}}_{k-1}^{\prime }\right)+1}{\left(2^{n_{k}-c_{k}}-1\right)q\left({\bar{s}}_{k-1}\right)+1}\leq 1. \end{array}$$

 □

**Lemma** **A6.**
*If $\left\{x_{t-1}=s\right\}=\left\{x_{t-1}^{\prime }=s^{\prime },x_{i,j,t-1}=0\right\}$, then $q\left(s^{\prime }\right)\geq q\left(s\right)$.*

**Proof.** The proof is given by induction. We, first, consider the case where k=2. Assume (without loss of generality) that the additional element is from the first conjunction ($c_{1}=c_{1}^{\prime }+1$, $c_{2}=c_{2}^{\prime }$). If $\min(s_{1}^{\prime })=0$, then $q\left(s^{\prime }\right)=q\left(s\right)$. If $\min(s_{2}^{\prime })=0$ and $\min(s_{1}^{\prime })=1$, then

$$q\left(s^{\prime }\right)=\frac{1}{2^{n_{1}-c_{1}^{\prime }}}>0=q\left(s\right).$$

Finally, if $\min\left(s_{1}^{\prime }\right)=\min\left(s_{2}^{\prime }\right)=1$, then

$$\frac{q(s^{\prime })}{q(s)}=\frac{2^{n_{2}-c_{2}^{\prime }}\left(2^{n_{1}-c_{1}^{\prime }}+2^{n_{2}-c_{2}^{\prime }}-1\right)}{2^{n_{1}+n_{2}-c_{1}^{\prime }-c_{2}^{\prime }}}=\frac{2^{n_{1}-c_{1}^{\prime }}+2^{n_{2}-c_{2}^{\prime }}-1}{2^{n_{1}-c_{1}^{\prime }}}\geq 1.$$

Therefore, when k=2, we have that $q\left(s^{\prime }\right)\geq q\left(s\right)$. Next, we assume the result holds for k−1, $q\left({\bar{s}}_{k-1}^{\prime }\right)\geq q\left({\bar{s}}_{k-1}\right)$ and demonstrate the result for general *k*. Again, assume the additional element is from the first conjunction ($c_{1}=c_{1}^{\prime }+1,c_{j}=c_{j}^{\prime }$ for j>1). If $\min(s_{k})=0$, then

$$\frac{q({\bar{s}}_{k}^{\prime })}{q({\bar{s}}_{k})}=\frac{q({\bar{s}}_{k-1}^{\prime })}{q({\bar{s}}_{k-1})}\leq 1,$$

and, if $\min(s_{k})=1$, then

$$\begin{array}{ccc} \frac{q({\bar{s}}_{k}^{\prime })}{q({\bar{s}}_{k})} & = & \frac{q\left({\bar{s}}_{k-1}^{\prime }\right)+\left(1-q\left({\bar{s}}_{k-1}^{\prime }\right)\right)/2^{n_{k}-c_{k}}}{q\left({\bar{s}}_{k-1}\right)+\left(1-q\left({\bar{s}}_{k-1}\right)\right)/2^{n_{k}-c_{k}}}\\ & = & \frac{\left(2^{n_{k}-c_{k}}-1\right)q\left({\bar{s}}_{k-1}^{\prime }\right)+1}{\left(2^{n_{k}-c_{k}}-1\right)q\left({\bar{s}}_{k-1}\right)+1}\geq 1. \end{array}$$

 □

Next, we again consider a normalization term Q(c), which is the sum of q(s) over all states of the occurrence. Here, we demonstrate the effect on Q(c) of adding an additional element to an occurrence:
**Lemma** **A7.** For an occurrence $\left\{X_{t-1}=x_{t-1}\right\}$ with $|x_{t-1}|=c>0$, define $Q\left(c\right)=\sum_{s}q\left(s\right)$. Now, consider adding a single element to an occurrence, $x_{t-1}^{\prime }=\left\{x_{t-1},x_{i,j_{1},t-1}\right\}$, ($x_{i,j_{1},t-1}\notin x_{t-1}$), such that $c_{j_{1}}^{\prime }=c_{j_{1}}+1$ and $c_{j}^{\prime }=c_{j}$ for $j\neq j_{1}$; so that $c^{\prime }=c+1$. Then, $\frac{Q(c^{\prime })}{Q(c)}=2$.

**Proof.** The proof is again given by induction. We, first, consider the case where k=2,

$$\begin{array}{ccc} Q(c) & = & \sum_{s}q\left(s\right)\\ & = & \frac{2^{c_{1}}-1}{2^{n_{2}-c_{2}}}+\frac{2^{c_{2}}-1}{2^{n_{1}-c_{1}}}+\frac{2^{n_{1}-c_{1}}+2^{n_{2}-c_{2}}-1}{2^{n_{1}+n_{2}-c_{1}-c_{2}}}\\ & = & \frac{2^{n_{1}}+2^{n_{2}}-1}{2^{n_{1}+n_{2}-c_{1}-c_{2}}} \end{array}$$

Assume (without loss of generality) that the additional element to the first conjunction ($c_{1}^{\prime }=c_{1}+1$). Then, we have that

$$\frac{Q(c^{\prime })}{Q(c)}=\frac{2^{n_{1}+n_{2}-c_{1}-c_{2}}\left(2^{n_{1}}+2^{n_{2}}-1\right)}{2^{n_{1}+n_{2}-c_{1}^{\prime }-c_{2}^{\prime }}\left(2^{n_{1}}+2^{n_{2}}-1\right)}=\frac{2^{n_{1}+n_{2}-c_{1}-c_{2}}}{2^{n_{1}+n_{2}-\left(c_{1}+1\right)-c_{2}}}=2.$$

Therefore, when k=2, we have that $\frac{Q(c^{\prime })}{Q(c)}=2$. Next, we assume the result holds for k−1 and demonstrate the result for general *k*. Using the recursive relationship for *q*, we get

$$\begin{array}{ccc} Q_{k}\left(c\right) & = & \sum_{{\bar{s}}_{k}}q\left({\bar{s}}_{k}\right)\\ & = & \sum_{s_{k}}\sum_{{\bar{s}}_{k-1}}q\left({\bar{s}}_{k-1},s_{k}\right)\\ & = & \left(2^{c_{k}}-1\right)\sum_{{\bar{s}}_{k-1}}q\left({\bar{s}}_{k-1}\right)+\sum_{{\bar{s}}_{k-1}}\left(q\left({\bar{s}}_{k-1}\right)+\frac{\left(1-q\left({\bar{s}}_{k-1}\right)\right)}{2^{n_{k}-c_{k}}}\right)\\ & = & \frac{\left(2^{n_{k}}-1\right)Q_{k-1}\left(c-c_{k}\right)+2^{c-c_{k}}}{2^{n_{k}-c_{k}}}. \end{array}$$

Again, assuming that the additional element is from the first conjunction $c_{1}^{\prime }=c_{1}+1$, for the ratio we have

$$\begin{array}{ccc} \frac{Q_{k}\left(c^{\prime }\right)}{Q_{k}\left(c\right)} & = & \frac{\left(2^{n_{k}}-1\right)Q_{k-1}\left(c^{\prime }-c_{k}^{\prime }\right)+2^{c^{\prime }-c_{k}^{\prime }}}{\left(2^{n_{k}}-1\right)Q_{k-1}\left(c-c_{k}\right)+2^{c-c_{k}}}\\ & = & \frac{\left(2^{n_{k}}-1\right)2Q_{k-1}\left(c-c_{k}\right)+2^{(c-c_{k})+1}}{\left(2^{n_{k}}-1\right)Q_{k-1}\left(c-c_{k}\right)+2^{c-c_{k}}}\\ & = & 2\left(\frac{\left(2^{n_{k}}-1\right)Q_{k-1}\left(x_{t-1}^{\prime }\right)+2^{c-c_{k}}}{\left(2^{n_{k}}-1\right)Q_{k-1}\left(x_{t-1}^{\prime }\right)+2^{c-c_{k}}}\right)\\ & = & 2. \end{array}$$

 □

The final two Lemmas demonstrate conditions under which the probability of $\left\{Y_{t}=1\right\}$ is either strictly increasing or strictly decreasing.

**Lemma** **A8.**
*If $\min(x_{t-1})=1$, $c_{j}<n_{j}\;\forall \;j$ and $x_{t-1}^{\prime }\subset x_{t-1}$, then $q\left(s^{\prime }\right)<q\left(s\right)$.*

**Proof.** The proof is given by induction. We, first, consider the case where k=2. Assume (without loss of generality) that $x_{t-1}$ has an additional element in the first conjunction, relative to $x_{t-1}^{\prime }$ ($c_{1}=c_{1}^{\prime }+1$, $c_{2}=c_{2}^{\prime }$). The result can be applied recursively for differences of more than one element:

$$\begin{array}{ccc} \frac{q(s)}{q(s^{\prime })} & = & \left(\frac{2^{n_{1}-c_{1}}+2^{n_{2}-c_{2}}-1}{2^{n_{1}-c_{1}^{\prime }}+2^{n_{2}-c_{2}^{\prime }}-1}\right)\left(\frac{2^{n_{1}+n_{2}-c_{1}^{\prime }-c_{2}^{\prime }}}{2^{n_{1}+n_{2}-c_{1}-c_{2}}}\right)\\ & = & 2\left(\frac{2^{n_{1}-c_{1}}+2^{n_{2}-c_{2}}-1}{2^{n_{1}-c_{1}+1}+2^{n_{2}-c_{2}}-1}\right)\\ & > & 1\;(\sin\mathrm{ce}\;c_{2}<n_{2}). \end{array}$$

Therefore, when k=2, we have that $q\left(s^{\prime }\right)<q\left(s\right)$. Next, we assume the result holds for k−1, $q\left({\bar{s}}_{k-1}^{\prime }\right)<q\left({\bar{s}}_{k-1}\right)$ and demonstrate the result for general *k*. Again, assume that $x_{t-1}$ and $x_{t-1}^{\prime }$ differ by a single element in the first conjunction ($c_{1}=c_{1}^{\prime }+1,c_{j}=c_{j}^{\prime }$ for j>1). As $\min(s_{k})=1$,

$$\begin{array}{ccc} \frac{q({\bar{s}}_{k})}{q({\bar{s}}_{k}^{\prime })} & = & \frac{q\left({\bar{s}}_{k-1}^{\prime }\right)+\left(1-q\left({\bar{s}}_{k-1}^{\prime }\right)\right)/2^{n_{k}-c_{k}}}{q\left({\bar{s}}_{k-1}\right)+\left(1-q\left({\bar{s}}_{k-1}\right)\right)/2^{n_{k}-c_{k}}}\\ & = & \frac{\left(2^{n_{k}-c_{k}}-1\right)q\left({\bar{s}}_{k-1}^{\prime }\right)+1}{\left(2^{n_{k}-c_{k}}-1\right)q\left({\bar{s}}_{k-1}\right)+1}\\ & > & 1. \end{array}$$

 □

**Lemma** **A9.**
*If $\max(x_{t-1})=0$, $c_{j}\leq 1\;\forall \;j$ and $x_{t-1}^{\prime }\subset x_{t-1}$, then $q\left(s\right)<q\left(s^{\prime }\right)$.*

**Proof.** The proof is given by induction. We, first, consider the case where k=2. Assume (without loss of generality) that $x_{t-1}$ has an additional element in the first conjunction, relative to $x_{t-1}^{\prime }$ ($c_{1}=c_{1}^{\prime }+1=1$, $c_{2}=c_{2}^{\prime }$). The result can be applied recursively for differences of more than one element. First, consider the case where $c_{2}=1$. Then, we have

$$q\left(s^{\prime }\right)=\frac{1}{2^{n_{2}-c_{2}}}>0=q\left(s\right).$$

Next, consider the case where $c_{2}=0$:

$$q\left(s^{\prime }\right)=\frac{2^{n_{1}}+2^{n_{2}}-1}{2^{n_{1}+n_{2}}}=\frac{1}{2^{n_{2}}}\left(\frac{2^{n_{1}}+2^{n_{2}}-1}{2^{n_{1}}}\right)=q\left(s\right)\left(\frac{2^{n_{1}}+2^{n_{2}}-1}{2^{n_{1}}}\right)>q\left(s\right).$$

Therefore, when k=2, we have that $q\left(s\right)<q\left(s^{\prime }\right)$. Next, we assume the result holds for k−1, $q\left({\bar{s}}_{k-1}\right)<q\left({\bar{s}}_{k-1}^{\prime }\right)$, and demonstrate the result for general *k*. Again, assume that $x_{t-1}$ and $x_{t-1}^{\prime }$ differ by a single element in the first conjunction ($c_{1}=c_{1}^{\prime }+1,c_{j}=c_{j}^{\prime }$ for j>1). As $\min(s_{k})=0$,

$$\frac{q({\bar{s}}_{k})}{q({\bar{s}}_{k}^{\prime })}=\frac{q({\bar{s}}_{k-1})}{q({\bar{s}}_{k-1}^{\prime })}<1.$$

 □

Using the above Lemmas, we are now in a position to prove the actual causes and actual effects in the causal account of a single DOC and its inputs. We separately consider the case where the DOC is in the ‘ON’ and the ‘OFF’ state.

**Theorem** **A2.**
*Consider a dynamical causal network $G_{u}$, such that $V_{t}=\left\{Y_{t}\right\}$ is a DOC element that is a disjunction of k conditions, each of which is a conjunction of $n_{j}$ inputs, and $V_{t-1}={\left\{{\left\{V_{i,j,t-1}\right\}}_{i=1}^{n_{j}}\right\}}_{j=1}^{k}$ is the set of its $n=\sum_{j}n_{j}$ inputs. For a transition $v_{t-1}≺v_{t}$, the following hold:*

1. *If $y_{t}=1$,*
  (a) *The actual cause of $\left\{Y_{t}=1\right\}$ is an occurrence $\left\{X_{t-1}=x_{t-1}\right\}$, where $x_{t-1}={\left\{x_{i,j,t-1}\right\}}_{i=1}^{n_{j}}\subseteq v_{t-1}$, such that $\min(x_{t-1})=1$; and*
  (b) *The actual effect of $\left\{X_{t-1}=x_{t-1}\right\}$ is $\left\{Y_{t}=1\right\}$, if $\min(x_{t-1})=1$ and $|x_{t-1}|=c_{j}=n_{j}$; otherwise, $x_{t-1}$ is reducible.*
2. *If $y_{t}=0$,*
  (a) *The actual cause of $\left\{Y_{t}=0\right\}$ is an occurrence $x_{t-1}\subseteq v_{t-1}$, such that $\max(x_{t-1})=0$ and $c_{j}=1\;\forall \;j$; and*
  (b) *If $\max(x_{t-1})=0$ and $c_{j}\leq 1\;\forall \;j$, then the actual effect of $\left\{X_{t-1}=x_{t-1}\right\}$ is $\left\{Y_{t}=0\right\}$; otherwise, $x_{t-1}$ is reducible.*

**Proof.** **Part 1a:** The actual cause of $\left\{Y_{t}=1\right\}$. For an occurrence $\left\{X_{t-1}=x_{t-1}\right\}$, the probability of $x_{t-1}$ in the cause repertoire of $y_{t}$ is

$$\pi \left(x_{t-1}|y_{t}\right)=\frac{q(s)}{Q(c)}.$$

As $Y_{t}$ is a first-order occurrence, there is only one possible partition, and the causal strength of a potential link is, thus,

$$\alpha_{c}\left(x_{t-1},y_{t}\right)=\log_{2}\left(\frac{\pi (x_{t-1}|y_{t})}{\pi (x_{t-1})}\right)=\log_{2}\left(\frac{2^{c}q\left(s\right)}{Q(c)}\right)=\log_{2}\left(Q_{1}q\left(s\right)\right),$$

where $Q_{1}=\frac{2^{c}}{Q(c)}\;\forall \;c$ (by Lemma A7). If we, then, consider adding a single element to the occurrence $x_{t-1}^{\prime }=\left\{x_{t-1},x_{i,j,t-1}^{\prime }\right\}$ ($x_{i,j,t-1}^{\prime }\notin x_{t-1}$) then the difference in causal strength is

$$\alpha_{c}\left(x_{t-1},y_{t}\right)-\alpha_{c}\left(x_{t-1}^{\prime },y_{t}\right)=\log_{2}\left(\frac{Q_{1}\;q\left(s\right)}{Q_{1}\;q\left(s^{\prime }\right)}\right)=\log_{2}\left(\frac{q(s)}{q(s^{\prime })}\right).$$

Combining the above with Lemma A6, adding an element $x_{i,j,t-1}=0$ to an occurrence cannot increase the causal strength and, thus, occurrences that include elements in state ‘OFF’ cannot be the actual cause of $y_{t}$. By Lemma A5, adding an element $x_{i,j,t-1}=1$ to an occurrence cannot decrease the causal strength. Furthermore, if $c_{j}=n_{j}$ and $\min({\bar{x}}_{j,t-1})=1$, then q(s)=1 and

$$\alpha_{c}\left(y_{t},x_{t-1}\right)=\log_{2}\left(Q_{1}q\left(s\right)\right)=\log_{2}\left(Q_{1}\right),$$

independent of the number of elements in the occurrence from other conjunctions $c_{j^{\prime }}$ and their states $s_{j^{\prime }}$ ($j^{\prime }\neq j$). As the value $Q_{1}$ does not depend on the specific value of *j*, it must be the case that this is the maximum value of causal strength, $\alpha^{\max}\left(y_{t}\right)$. Furthermore, if $c_{j}<n_{j}\;\forall \;j$, then

$$\alpha_{c}\left(y_{t},x_{t-1}\right)=\log_{2}\left(Q_{1}q\left(s\right)\right)<\log_{2}\left(Q_{1}\right).$$

Therefore, the maximum value of causal strength is

$$\log_{2}\left(Q_{1}\right),$$

and an occurrence $x_{t-1}$ achieves this value (satisfying condition (1) of being an actual cause) if and only if there exists *j*, such that $c_{j}=n_{j}$ and $\min({\bar{x}}_{j,t-1})=1$ (i.e., the occurrence includes a conjunction whose elements are all ‘ON’). Consider an occurrence that satisfies condition (1), such that there exists $j_{1}$ with $c_{j_{1}}=n_{j_{1}}$. If there exists $j_{2}\neq j_{1}$ such that $c_{j_{2}}>0$, then we can define a subset $x_{t-1}^{\prime }\subset x_{t-1}$ with $c_{j_{1}}^{\prime }=n_{j_{1}}$ and $c_{j_{2}}^{\prime }=0$ that also satisfies condition (1) and, thus, $x_{t-1}$ does not satisfy condition (2). Finally, if no such $j_{2}$ exists ($x_{t-1}={\bar{x}}_{j,t-1}$), then any subset $x_{t-1}^{\prime }\subset x_{t-1}$ has $c_{j}<n_{j}\;\forall j$ and does not satisfy condition (1), so $x_{t-1}$ satisfies condition (2). Therefore, we have that the actual cause of $y_{t}$ is an occurrence $x_{t-1}={\bar{x}}_{j,t-1}$, such that $\min x_{t-1}=1$,

$$x^{*}\left(y_{t}\right)=\left\{{\bar{x}}_{j,t-1}\subset v_{t-1}|\min{\bar{x}}_{j,t-1}=1\right\}.$$

**Part 1b:** Actual effect of $x_{t-1}$ when $y_{t}=1$. Again, consider occurrences $X_{t-1}=x_{t-1}$ with $c_{j}$ elements from each of the *k* conjunctions. The effect repertoire of a DOC with *k* conjunctions over such occurrences is

$$\pi \left(y_{t}|x_{t-1}=s\right)=q\left(s\right).$$

As there is only one element in $v_{t}$, the only question is whether or not $x_{t-1}$ is reducible. If it is reducible, it has no actual effect; otherwise, its actual effect must be $y_{t}$. First, if there exists $x\in x_{t-1}$ with x=0, then we can define $x_{t-1}^{\prime }$ such that $x_{t-1}=\left\{x_{t-1}^{\prime },x\right\}$, and a partition $\psi =\left\{\left\{x_{t-1}^{\prime },y_{t}\right\},\left\{x,⌀\right\}\right\}$ (i.e., cutting away *x*), such that

$$\pi {\left(y_{t}|x_{t-1}\right)}_{\psi }=\pi \left(y_{t}|x_{t-1}^{\prime }\right)\times \pi \left(⌀|x\right)=\pi \left(y_{t}|x_{t-1}^{\prime }\right)=q\left(s^{\prime }\right).$$

By Lemma A6, $q\left(s^{\prime }\right)\geq q\left(s\right)$ and, thus,

$$\alpha_{e}\left(x_{t-1},y_{t}\right)\leq \log_{2}\left(\frac{\pi (y_{t}|x_{t-1})}{\pi {\left(y_{t}|x_{t-1}\right)}_{\psi }}\right)=\log_{2}\left(\frac{q(s)}{q(s^{\prime })}\right)\leq 0,$$

and so $x_{t-1}$ is reducible. Next, we consider the case where $\min(x_{t-1})=1$, but there exists $j_{1},j_{2}$ such that $c_{j_{1}}=n_{j_{1}}$ and $c_{j_{2}}>0$. We define $x_{t-1}^{\prime }={\bar{x}}_{j_{1},t-1}$ and a partition $\psi =\left\{\left\{x_{t-1}^{\prime },y_{t}\right\},\left(x_{t-1}\backslash x_{t-1}^{\prime }\right),⌀\}\right\}$, such that

$$\pi {\left(y_{t}|x_{t-1}\right)}_{\psi }=\pi \left(y_{t}|x_{t-1}^{\prime }\right)\times \pi \left(⌀|\left(x_{t-1}-x_{t-1}^{\prime }\right)\right)=\pi \left(y_{t}|x_{t-1}^{\prime }\right)=q\left(s^{\prime }\right),$$

and, thus,

$$\alpha_{e}\left(x_{t-1},y_{t}\right)\leq \log_{2}\left(\frac{\pi (y_{t}|x_{t-1})}{\pi {\left(y_{t}|x_{t-1}\right)}_{\psi }}\right)=\log_{2}\left(\frac{q(s)}{q(s^{\prime })}\right)=\log_{2}\left(\frac{1}{1}\right)=0,$$

so that $x_{t-1}$ is, again, reducible. We, now, split the irreducible occurrences into two cases. First, we consider $\min(x_{t-1})=1$ and all $c_{j}<n_{j}$. All possible partitions of the pair of occurrences can be formulated as $\psi =\left\{\left\{x_{t-1}^{\prime },y_{t}\right\},\left\{\left(x_{t-1}\backslash x_{t-1}^{\prime }\right),⌀\right\}\right\}$ (where $x_{t-1}^{\prime }\subset x_{t-1}$), such that

$$\pi {\left(y_{t}|x_{t-1}\right)}_{\psi }=\pi \left(y_{t}|x_{t-1}^{\prime }\right))\times \pi (⌀|\left(x_{t-1}-x_{t-1}^{\prime }\right)=\pi \left(y_{t}|x_{t-1}^{\prime }\right)=q\left(s^{\prime }\right),$$

and, by Lemma A8,

$$\alpha_{e}\left(x_{t-1},y_{t}\right)=\min_{\psi }\left(\log_{2}\left(\frac{\pi (y_{t}|x_{t-1})}{\pi {\left(y_{t}|x_{t-1}\right)}_{\psi }}\right)\right)=\min_{\psi }\left(\log_{2}\left(\frac{q(s)}{q(s^{\prime })}\right)\right)>0.$$

So, $x_{t-1}$ is irreducible, and its actual effect is $\left\{Y_{1}=1\right\}$. Next, we consider occurrences such that $\min(x_{t-1})=1$, $c_{j_{1}}=n_{j_{1}}$, and $c_{j}=0$ for $j\neq j_{1}$ (i.e., $x_{t-1}={\bar{x}}_{j_{1},t-1}$). All possible partitions of the pair of occurrences can be formulated as $\psi =\left\{\left\{x_{t-1}^{\prime },y_{t}\right\},\{\left(x_{t-1}-x_{t-1}^{\prime },⌀\right\}\right\}$ (where $x_{t-1}^{\prime }\subset x_{t-1}$), such that

$$\pi {\left(y_{t}|x_{t-1}\right)}_{\psi }=\pi \left(y_{t}|x_{t-1}^{\prime }\right)\times \pi (⌀|\left(x_{t-1}-x_{t-1}^{\prime }\right)=\pi \left(y_{t}|x_{t-1}^{\prime }\right)=q\left(s^{\prime }\right),$$

$$\alpha_{e}\left(x_{t-1},y_{t}\right)\leq \log_{2}\left(\frac{\pi (y_{t}|x_{t-1})}{\pi {\left(y_{t}|x_{t-1}\right)}_{\psi }}\right)=\log_{2}\left(\frac{q(s)}{q(s^{\prime })}\right)=\log_{2}\left(\frac{1}{q(s^{\prime })}\right)>0,$$

and $x_{t-1}$ is, again, irreducible with actual effect $\left\{Y_{t}=1\right\}$. **Part 2a:** The actual cause of $\left\{Y_{t}=0\right\}$. For an occurrence $\left\{X_{t-1}=x_{t-1}\right\}$, the cause repertoire of $y_{t}$ is

$$\pi \left(x_{t-1}|y_{t}\right)=\frac{1-q(s)}{2^{c}-Q\left(c\right)}.$$

As $Y_{t}$ is a first-order occurrence, there is only one possible partition, and the causal strength of a potential link is, thus,

$$\alpha_{c}\left(x_{t-1},y_{t}\right)=\log_{2}\left(\frac{\pi (x_{t-1}|y_{t})}{\pi (x_{t-1})}\right)=\log_{2}\left(\frac{2^{c}\left(1-q\left(s\right)\right)}{2^{c}-Q\left(c\right)}\right)=\log_{2}\left(Q_{0}\;q\left(s\right)\right),$$

where $Q_{0}=\frac{2^{c}}{2^{c}-Q\left(c\right)}\;\forall \;c$ (Lemma A7). If we, then, consider adding a single element to the occurrence $x_{t-1}^{\prime }=\left\{x_{t-1},x_{i,j,t-1}^{\prime }\right\}$ ($x_{i,j,t-1}^{\prime }\notin x_{t-1}$), then the difference in causal strength is

$$\alpha_{c}\left(x_{t-1},y_{t}\right)-\alpha_{c}\left(x_{t-1}^{\prime },y_{t}\right)=\log_{2}\left(\frac{Q_{0}\left(1-q\left(s\right)\right)}{Q_{0}\left(1-q\left(s^{\prime }\right)\right)}\right)=\log_{2}\left(\frac{1-q(s)}{1-q(s^{\prime })}\right).$$

By Lemma A6, adding an element x=1 to an occurrence cannot increase the causal strength and, thus, occurrences that include elements in the state ‘ON’ cannot be the actual cause of $y_{t}$. By Lemma A5, adding an element x=0 to an occurrence cannot decrease the causal strength. If $c_{j}>0\;\forall \;j$ and $\max(x_{t-1})=0$, then

$$\alpha_{c}\left(y_{t},x_{t-1}\right)=\log_{2}\left(Q_{0}\left(1-q\left(s\right)\right)\right)=\log_{2}\left(Q_{0}\right),$$

independent of the actual values of $c_{j}$. As this holds for any set of $c_{j}$ that satisfies the conditions, it must be the case that this value is $\alpha^{\max}\left(y_{t}\right)$. Furthermore, if there exists *j* such that $c_{j}=0$, then

$$\alpha_{c}\left(y_{t},x_{t-1}\right)=\log_{2}\left(Q_{0}\left(1-q\left(s\right)\right)\right)<\log_{2}\left(Q_{0}\right).$$

Therefore, the maximum value of causal strength is

$$\log_{2}\left(Q_{0}\right),$$

and an occurrence $x_{t-1}$ achieves this value (satisfying condition (1) of being an actual cause) if and only if $c_{j}>0\;\forall \;j$ and $\max(x_{t-1})=0$ (i.e., the occurrence contains elements from every conjunction, and only elements whose state is ‘OFF’). Consider an occurrence $x_{t-1}$ that satisfies condition (1). If there exists $j_{1}$ such that $c_{j_{1}}>1$, then we can define a subset $x_{t-1}^{\prime }\subset x_{t-1}$ with $c_{j_{1}}^{\prime }=1$ that also satisfies condition (1) and, thus, $x_{t-1}$ does not satisfy condition (2). Finally, if $c_{j}=1\;\forall \;j$ then for any subset $x_{t-1}^{\prime }\subset x_{t-1}$ there exists *j* such that $c_{j}^{\prime }=0$, so $x_{t-1}^{\prime }$ does not satisfy condition (1) and, thus, $x_{t-1}$ satisfies condition (2). Therefore, we have that the actual cause of $y_{t}$ is an occurrence $x_{t-1}$, such that $\max x_{t-1}=0$ and $c_{j}=1\;\forall \;j$,

$$x^{*}\left(y_{t}\right)=\left\{x_{t-1}\subseteq v_{t-1}|\max\left(x_{t-1}\right)=0\;\mathrm{and}\;c_{j}=1\;\forall \;j\right\}.$$

**Part 2b:** Actual effect of $x_{t-1}$ when $y_{t}=0$. Again, consider occurrences $X_{t-1}=x_{t-1}$ with $c_{j}$ elements from each of *k* conjunctions. The probability of $y_{t}$ in the effect repertoire of $x_{t-1}$ is

$$\pi \left(y_{t}|x_{t-1}=s\right)=1-q\left(s\right).$$

As there is only one element in $v_{t}$, the only question is whether or not $x_{t-1}$ is reducible. If it is reducible, it has no actual effect; otherwise, its actual effect must be $y_{t}$. First, if there exists $x_{i,j,t-1}\in x_{t-1}$ such that $x_{i,j,t-1}=1$, then we can define $x_{t-1}^{\prime }$ such that $x_{t-1}=\left\{x_{t-1}^{\prime },x_{i,j,t-1}\right\}$ and a partition $\psi =\left\{\left\{x_{t-1}^{\prime },y_{t}\right\},\left\{x_{i,j,t-1},⌀\right\}\right\}$, such that

$$\pi {\left(y_{t}|x_{t-1}\right)}_{\psi }=\pi \left(y_{t}|x_{t-1}^{\prime }\right)\times \pi \left(⌀|x_{i},j,t-1\right)=\pi \left(y_{t}|x_{t-1}^{\prime }\right)=1-q\left(s^{\prime }\right).$$

By Lemma A5, we have $1-q\left(s\right)\leq 1-q\left(s^{\prime }\right)$ and, thus,

$$\alpha_{e}\left(x_{t-1},y_{t}\right)\leq \log_{2}\left(\frac{\pi (y_{t}|x_{t-1})}{\pi {\left(y_{t}|x_{t-1}\right)}_{\psi }}\right)=\log_{2}\left(\frac{1-q(s)}{1-q(s^{\prime })}\right)\leq 0,$$

and so $x_{t-1}$ is reducible. Next, we consider the case where $\max(x_{t-1})=0$, but there exists *j* such that $c_{j}>1$. We define $x_{t-1}^{\prime }$ with $c_{j}^{\prime }=1\;\forall \;j$ such that $x_{t-1}=\left\{x_{t-1}^{\prime },x_{i,j,t-1}\right\}$, and a partition $\psi =\left\{\left\{x_{t-1}^{\prime },y_{t}\right\},\left\{x_{i,j,t-1},⌀\right\}\right\}$, such that

$$\pi {\left(y_{t}|x_{t-1}\right)}_{\psi }=\pi \left(y_{t}|x_{t-1}^{\prime }\right)\times \pi \left(⌀|x_{i,j,t-1}\right)=\pi \left(y_{t}|x_{t-1}^{\prime }\right)=q\left(s^{\prime }\right)=1$$

and

$$\alpha_{e}\left(x_{t-1},y_{t}\right)\leq \log_{2}\left(\frac{\pi (y_{t}|x_{t-1})}{\pi {\left(y_{t}|x_{t-1}\right)}_{\psi }}\right)=\log_{2}\left(\frac{1-q(s)}{1-q(s^{\prime })}\right)=\log_{2}\left(\frac{1}{1}\right)=0,$$

and so $x_{t-1}$ is, again, reducible. Finally, we show that the occurrences $x_{t-1}$ are irreducible if $\max(x_{t-1})=0$ and all $c_{j}\leq 1$. All possible partitions of the pair of occurrences can be formulated as $\psi =\left\{\left\{x_{t-1}^{\prime },y_{t}\right\},\left\{\left(x_{t-1}\backslash x_{t-1}^{\prime }\right),⌀\right\}\right\}$ (where $x_{t-1}^{\prime }\subset x_{t-1}$), such that $c_{j}^{\prime }\leq c_{j}\;\forall \;j$, and $c^{\prime }<c$. Then,

$$\pi {\left(y_{t}|x_{t-1}\right)}_{\psi }=\pi \left(y_{t}|x_{t-1}^{\prime }\right)\times \pi \left(⌀|\left(x_{t-1}-x_{t-1}^{\prime }\right)\right)=\pi \left(y_{t}|x_{t-1}^{\prime }\right)=1-q\left(s^{\prime }\right),$$

and, by Lemma A9,

$$\alpha_{e}\left(x_{t-1},y_{t}\right)=\min_{\psi }\left(\log_{2}\left(\frac{\pi (y_{t}|x_{t-1})}{\pi {\left(y_{t}|x_{t-1}\right)}_{\psi }}\right)\right)=\min_{\psi }\left(\log_{2}\left(\frac{1-q(s)}{1-q(s^{\prime })}\right)\right)>0.$$

Therefore, $\left\{X_{t-1}=x_{t-1}\right\}$ is irreducible, and its actual effect is $\left\{Y_{1}=1\right\}$. □
